# Analysis of pituitary adenoma expression patterns suggests a potential role for the NeuroD1 transcription factor in neuroendocrine tumor-targeting therapies

**DOI:** 10.18632/oncotarget.26513

**Published:** 2019-01-08

**Authors:** Lubov Borisovna Mitrofanova, Olga Mikhailovna Vorobeva, Andrey Nikolaevich Gorshkov

**Affiliations:** ^1^ Almazov National Medical Research Center, St. Petersburg, Russia; ^2^ Smorodintsev Research Institute of Influenza, St. Petersburg, Russia

**Keywords:** pituitary adenoma, NeuroD1 transcription factor, confocal laser scanning microscopy, electron immunocytochemistry

## Abstract

NeuroD1’s roles in the pathogenesis of pituitary adenomas and in the biology of the normal adult pituitary gland have been insufficiently researched. Much of the work investigating its expression patterns has yielded contradictory results. Objective: morphological study of NeuroD1 transcription factor expression in different types of pituitary adenomas and in normal adult human pituitary glands. Materials and methods: This study analyzed 48 pituitary adenomas and nine normal pituitary glands. In all cases, immunohistochemical study was performed with antibodies to NeuroD1, 6 hormones of adenohypophysis, Ki-67, and CK7. We used confocal laser scanning microscopy, electron microscopy and electron immunocytochemistry. Results: NeuroD1 expression was detected in all cases of plurihormonal adenomas, mammosomatotropinomas, corticotropinomas, prolactinomas, gonadotropinomas, null-cell pituitary adenomas, and in normal pituitary glands. The average numbers of NeuroD1 expressing cells in normal adenohypophysis specimens were significantly lower than in the adenomas overall (*p*=0.006). NeuroD1 expression was confirmed by several methods (in prolactinomas, by double stain immunohistochemistry; in mammosomatotropinomas, by double stain immunohistochemistry, confocal laser scanning microscopy, and electron immunocytochemistry; and in somatotropinomas, by electron immunocytochemistry). Conclusion: Immunohistochemistry, confocal microscopy, and double label electron immunocytochemistry confirmed NeuroD1’s key role in the pathogenesis of pituitary tumors, regardless of their hormonal state. Its expression level in pituitary adenomas is significantly higher than in the normal pituitary gland and has no reliable correlation with any studied hormones or Ki-67. These findings suggest that NeuroD1 should be investigated further as a potential molecular target in tumor-targeting therapies.

## INTRODUCTION

Pituitary adenomas comprise 15-20% of all intracranial tumors. They have various clinical manifestations depending on proliferative and hormonal activity. World Health Organization (WHO) guidelines for 2017 specify the following pituitary adenoma types: somatotroph adenomas (densely granulated somatotroph adenoma, sparsely granulated somatotroph adenoma, mammosomatotroph adenoma, mixed somatotroph-lactotroph adenoma); lactotroph adenomas (sparsely granulated lactotroph adenoma, densely granulated lactotroph adenoma, acidophil stem cell adenoma); thyrotroph adenoma; corticotroph adenomas (densely granulated corticotroph adenoma, sparsely granulated corticotroph adenoma, crooke’s cell adenoma); gonadotroph adenoma; null cell adenoma; plurihormonal adenomas (Pit-1-positive plurihormonal adenoma, previously termed *silent subtype 3 adenoma*); and adenomas with unusual immunohistochemical combinations [1].

The impacts of pituitary tumors are diverse, and symptoms range from nonexistent to severe. Pituitary tumors that secrete hormones (functioning) can cause a variety of signs and symptoms depending on the hormones they produce. Tumors that do not secrete hormones (nonfunctioning) cause signs and symptoms as result of their growth and impingement on other structures. Signs and symptoms of pituitary tumor pressure may include vision loss, headaches, and loss of peripheral vision in particular. Hormone dysregulation induced symptoms can include: menstrual changes; sexual dysfunction; elevated blood sugar or pressure; body weight changes; adipose distribution changes (accumulation at the midsection and upper back); thinning of the extremities; diuresis; weakness; nausea; vomiting; feeling cold; weakening of bones; cardiac problems; exaggerated facial features; depression, anxiety, or irritability (adrenocorticotropic hormone-secreting tumors); acne; misaligned teeth; increased body hair (growth hormone-secreting tumors); excess sweating; enlarged hands and feet; stretch marks; propensity to bruising; joint pain; and other symptoms [2].

Therefore, despite their classification as benign, pituitary adenomas can be clinically serious, and they often represent a significant burden. The main treatment method for adenoma is transsphenoidal resection, but they often recur following intervention. Surgical remission rates of up to 60% have been reported [3, 4]. Our continually improving understanding of pituitary cell molecular biology has allowed effective, targeted therapies to be developed. If pharmacotherapy is needed after surgery, dopamine agonists are recommended for adenomas that dual secrete growth hormone (GH) and prolactin because both expression pathways are targeted by these agents. In GH-secreting adenomas, somatostatin receptors (SSTR2 and SSTR5 subtypes) make up 90–95% of receptor expression. Lanreotide and octreotide are the main somatostatin analogs currently in use and they activate the signaling pathway to inhibit hormone production in functional adenomas [5, 6]. Targeted molecular therapy for acromegaly, using GH-receptor antagonists, represents a third example of success in terms of insights from molecular biology being translated into practice. The only GH-receptor antagonist approved by the FDA (United States Food and Drug Administration) available for the treatment of acromegaly is Pegvisomant. As a pegylated analog of human GH, it competes directly with plasma GH for receptor binding [7]. Despite progress with somatotropinoma, the search for targeted therapies which are effective with other pituitary adenoma types continues.

In recent years, our understanding of the cellular and molecular biology of pituitary gland tumors has changed significantly. It is known that transcription factors (TFs) regulate the differentiation of pituitary precursor cells into mature secretory cells during embryogenesis [8, 9]; those studies were performed mostly in animals. The relevant transcription factors are thought to be Prop-1, Pit-1, Pitx-1, NeuroD1, SF1, Gata2, RPx/Hes1, Pitx1, Рtx2, Lhx3/LIM3/P-Lim, and others. It is known that the NeuroD1 TF participates in corticotroph formation. Сorticotrophs are the first cells which differentiate in the developing pituitary gland [10]. In the NeuroD family, three isoforms have been identified: NeuroD1, NeuroD2, and NeuroD3. NeuroD1 and NeuroD2 are initially produced during embryogenesis and remain in the adult nervous system unlike NeuroD3, which is only briefly expressed in the 9-10th week of gestation. NeuroD family proteins are also expressed in primitive neuroectodermal tumors. NeuroD1 was found in pancreatic endocrine cells and was named BETA2 (b-cell trans-activator E-box 2). Mice with NeuroD1 mutations die soon after birth from severe neonatal diabetes. Its synergism with a different transcription factor, Pitx1, has also been described [11]. NeuroD1’s role in the pathogenesis of pituitary adenomas and in the biology of the normal adult pituitary gland has been insufficiently researched. Much of the work addressing its expression is contradictory. For example, it is understood that Neuro D1 participates in the formation of corticotrophs and is expressed in corticotropinomas, yet it has been seen in thyrotropinomas and null-cell pituitary adenomas. Collectively, these various patterns show that more work is needed to fully understand Neuro D1 [12].

NeuroD1 is known to play an important role in neuronal differentiation [13, 14]. Because of its importance during embryonic neurogenesis, it has been recently used in work aiming to reprogram other somatic cell types into becoming neurons. One study used a combination of factors (Pou3f2, Ascl1, Myt1l, NeuroD1) to successfully reprogram fetal and postnatal fibroblasts into neurons [15]. In addition, NeuroD1 alone was capable of converting reactive glial cells into functional neurons *in vivo*; it was also able to convert human astrocytes into glutamatergic neurons [16]. Some researchers (Pataskar et al.) have concluded that NeuroD1 is a powerful factor involved in neuron development [17]. In light of NeuroD1’s range of functions, and contradictory data on its expression in pituitary adenomas, it seems plausible to us that its role may not be limited to simply support of corticotroph formation.

**Objective**: morphological study of NeuroD1 transcription factor expression in different types of pituitary adenomas and in normal adult human pituitary glands.

## RESULTS

### Plurihormonal pituitary adenomas

**Figure 1:**
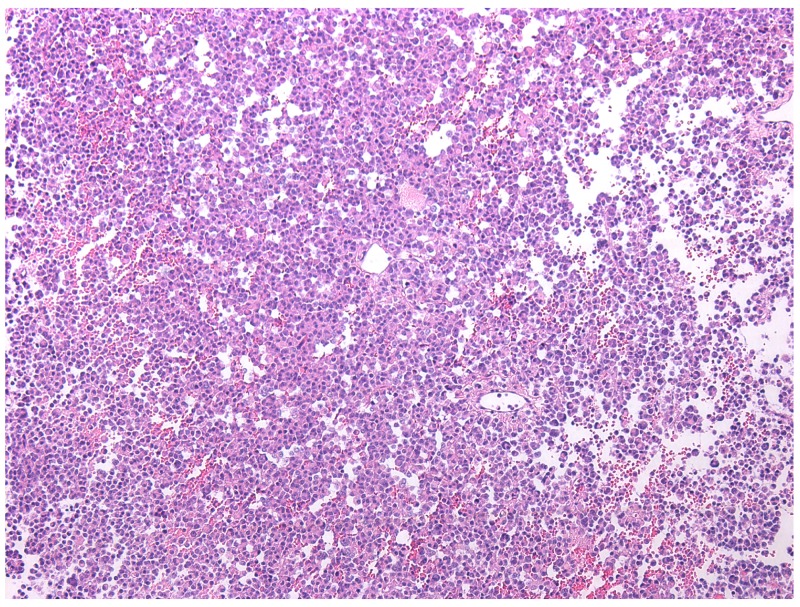
Plurihormonal pituitary adenoma with trabecular structure, hematoxylin and eosin, ×100

This group included clinically diagnosed somatotropinomas (two cases), corticotropinomas (two cases), prolactinoma (one case), and non-functioning pituitary adenomas (two cases). According to magnetic resonance imaging data, the average size of plurihormonal adenomas was 14х14х12 mm. The microscopic morphologies of plurihormonal pituitary adenomas were the most heterogeneous of all adenoma types. Solid, trabecular, papillary, and sinusoidal structures were seen in the tumors, sometimes in combination (Figure 1). They were PAS stain negative. In the plurihormonal adenomas, hormone expression (when present) was cytoplasmic, and NeuroD1 expression was nuclear. NeuroD1 expression was seen in all cases.

**Figure 2:**
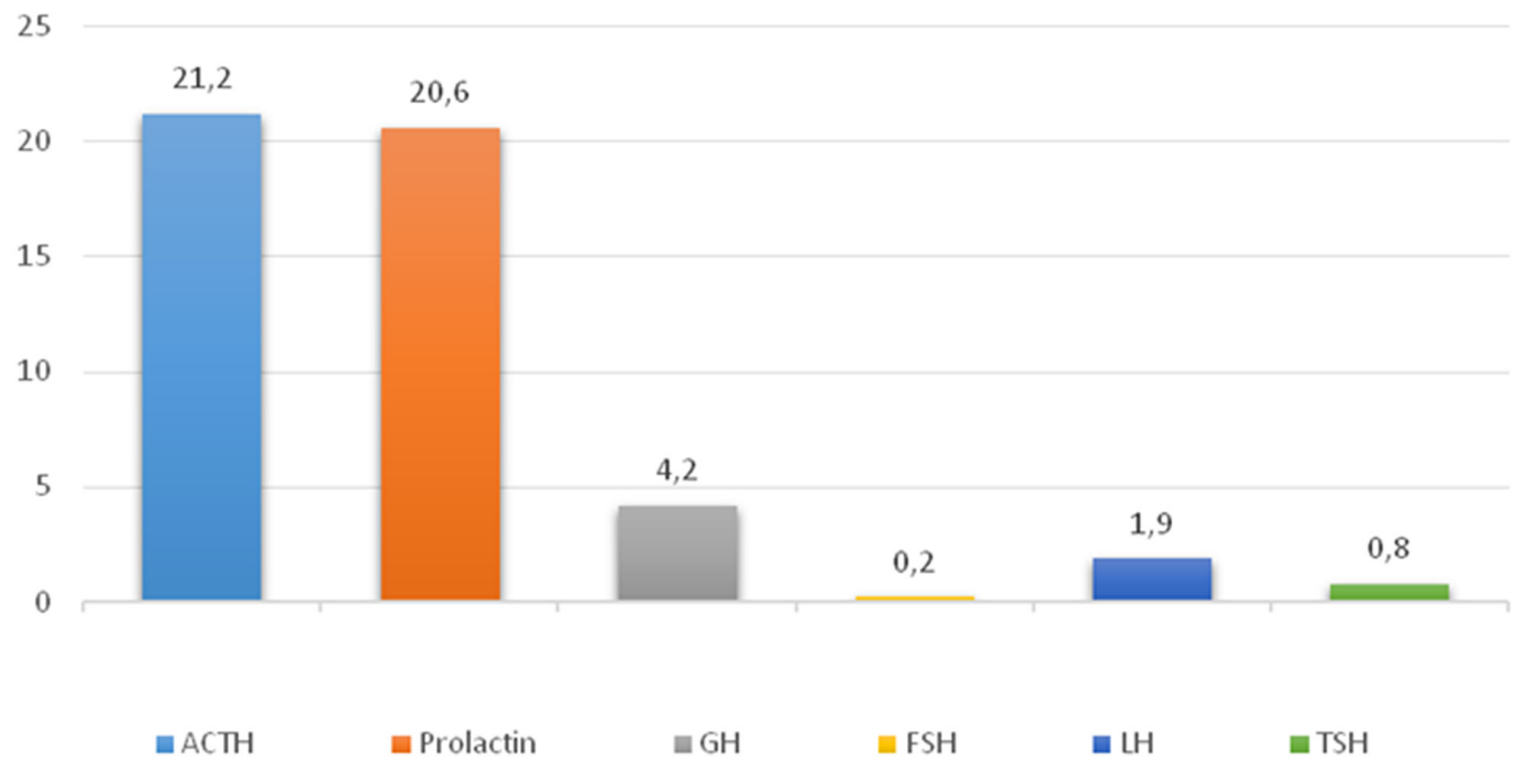
Average hormone expression values in plurihormonal pituitary adenomas Numbers indicate the average number of hormone expressing cells (percent).

**Figure 3:**
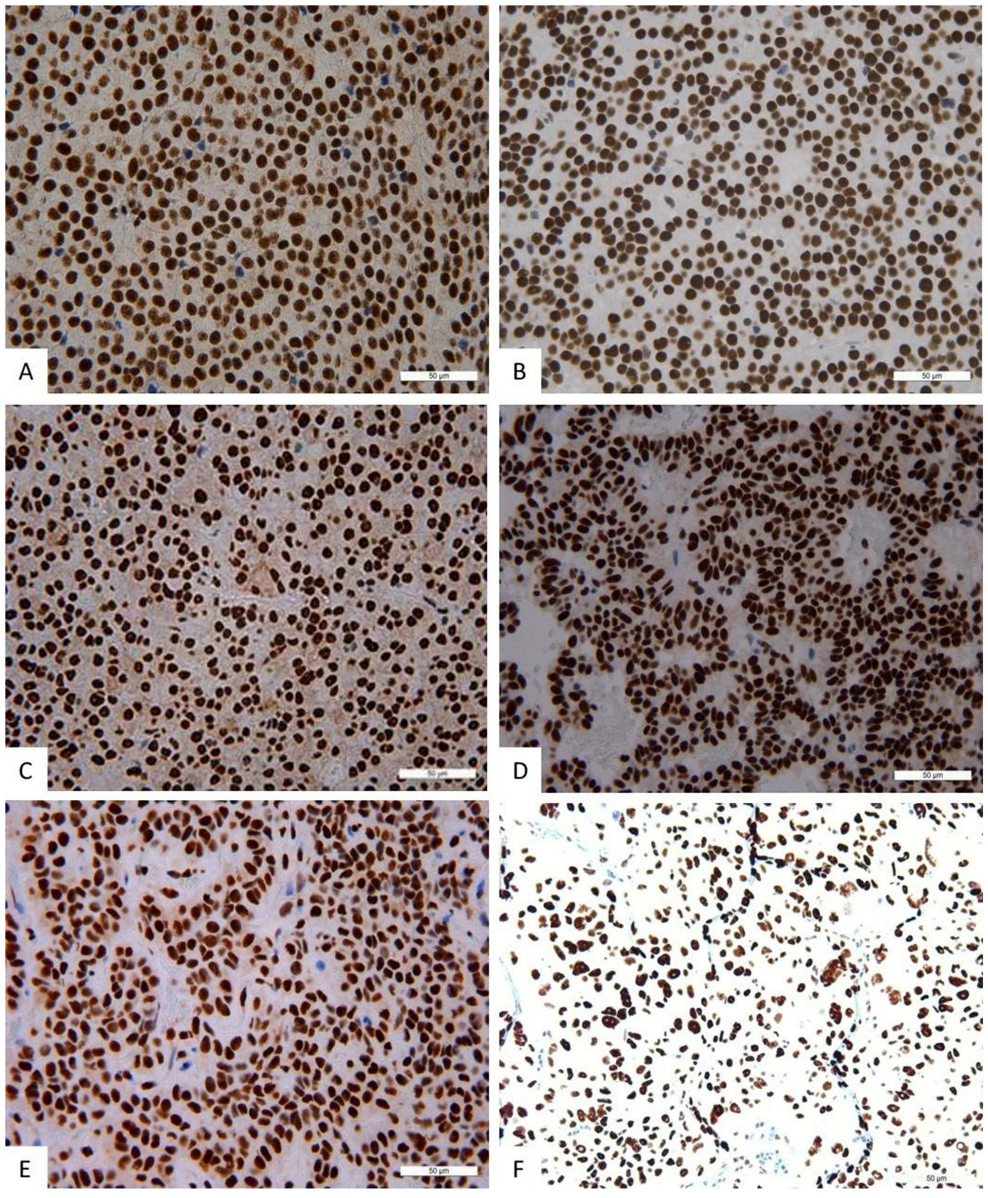
Immunohistochemistry, NeuroD1 in different pituitary adenomas Expression of Neuro D1 seen as brown staining of tumor cell nuclei; х200. **(A)** Plurihormonal adenoma. **(B)** Mammosomatotropinoma. **(C)** Prolactinoma. **(D)** Gonadotropinoma. **(E)** Null-cell adenoma. **(F)** Somatotropinoma.

**Figure 4:**
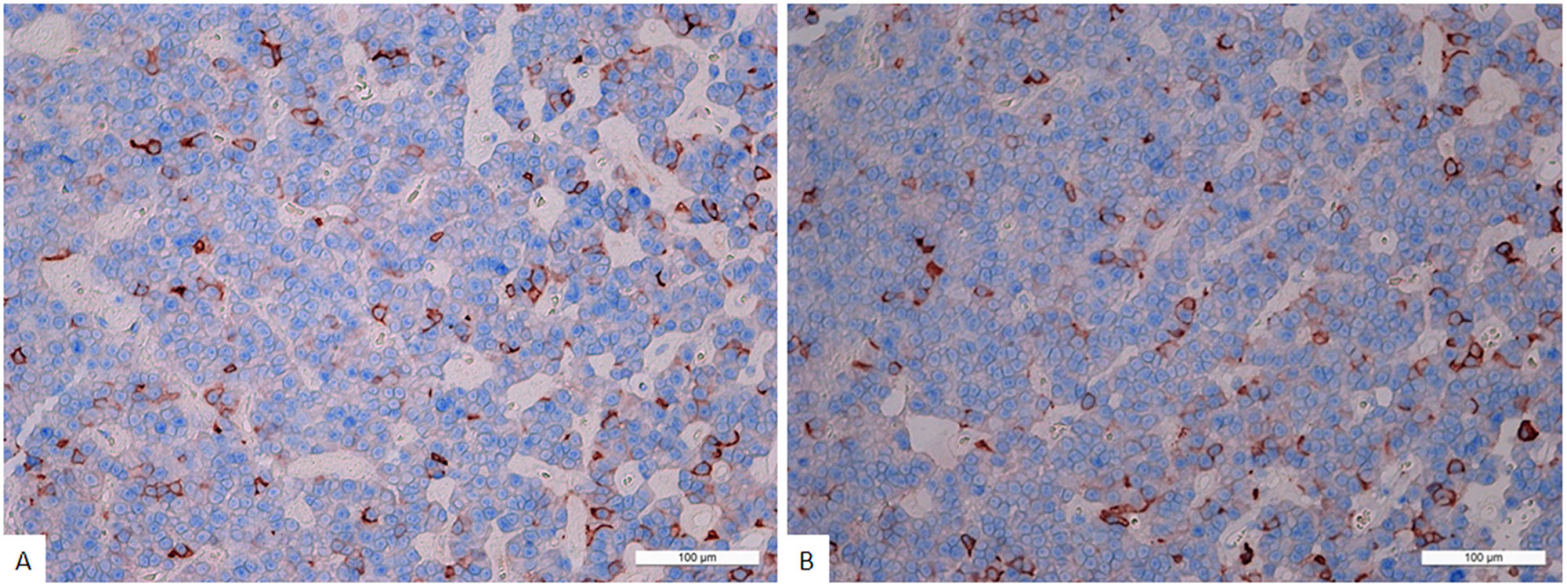
**(A)** Double stain immunohistochemistry, plurihormonal adenoma, Prolactin/NeuroD1, × 200. Prolactin is visualized with red colour, NeuroD1 with blue colour. Same cell co-expression of Prolactin and NeuroD1 is 11%. Generally, the average number of cells with co-expression (Prolactin/NeuroD1) in this pituitary adenoma is 11.3 ± 7.2%. **(B)** Double stain immunohistochemistry, plurihormonal adenoma, Growth hormone/NeuroD1, × 200. Growth hormone is visualized with red colour, NeuroD1 with blue colour. Same cell co-expression of Growth hormone/NeuroD1 is 12%. Generally, the average number of cells with co-expression (Growth hormone/NeuroD1) in this pituitary adenoma is 10.0 ± 3.1%.

The average numbers of cells expressing given markers were: ACTH 21.2±24.8% (from 5% to 76%); Prolactin 20.6±12.3% (from 3.8% to 45.1%); GH 4.2±3.6% (from 1.3% to 10%); FSH 0.2±0.3% (from 0% to 0.9%); LH 1.9±2.7% (from 0% to 7.2%); TSH 0.8±1.8% (from 0% to 5%; Figure 2); and NeuroD1 89.4±13.4% (from 69.9% to 99.5%; Figure 3, Table 1). The Ki-67 index was 1.4±1.2% (from 0.1% to 3.3%). Double stain immunohistochemistry revealed co-expression patterns. PRL and NeuroD1 were co-expressed in 5.2% of cells, on average (from 1.1 to 31.1%). GH and NeuroD1 were co-expressed in 4.6% of cells, on average (from 2.9 to 8.4%) (Figure 4A, B).

**Table 1:** Average hormone and NeuroD1 expression values

Sample	Average hormone and NeuroD1 expression values (in percent)						
	ACTH average min-max	PRL average min-max	GH average min-max	FSH average min-max	LH average min-max	TSH average min-max	NeuroD1 average min-max
Plurihormonal adenomas (n=7)	21.2±24.8 5-76	20.6±12.3 3.8-45.1	4.2±3.6 1.3-10	0.2±0.3 0-0.9	1.9±2.7 0-7.2	0.8±1.8 0-5	89.4±13.4 69.9-99.5
Null-cell adenomas (n=8)	0	0	0	0	0	0	94.1±5.8 85.1-98.8
Corticotropinomas (n=8)	51.1±17.4 21-78	0.7±0.7 0.1-1.8	0	0	0	0	94.9±4.1 87.8-99.7 ^*^
Prolactinomas (n=8)	0.05±0.08 0-0.2	45.8±5.6 40-55	0.03±0.04 0.04-0.1	0	0	0	98.9±0.6 98.2-99.3
Mammosomatotropinomas (n=10)	0	50.0±10.3 33.0-61.2	30.2±8.8 18.5-41.1	0	0	0	97.4±2.6 92.9-99.7 ^*^
Gonadotropinomas (n=5)	0	0	0	12.8±14.4 0.2-35.3	24.0±14.5 10.5-45.8	0	99.2±0.4 98.8-99.8 ^*^
Normal pituitary (n=9)	44.7±10.0 34.7-54.6	52.2±4.6 47.6-56.8	46.3±13.1 33.2-59.4	52.3±7.5 44.8-59.7	18.7±3.6 15.1-22.2	31.1±8.6 22.5-39.6	67.8±23.3 14-90 ^*^

^*^ statistically significant differences in NeuroD1 levels between normal pituitary and adenomas.

### Corticotropinomas

This group contained seven cases of clinically diagnosed corticotropinoma and one case of prolactinoma. According to magnetic resonance imaging data, the average size of corticotropinomas was 25х23х23 mm. Densely granulated corticotropinoma was diagnosed in five cases, and sparsely granulated corticotropinoma was diagnosed in three cases. Secretory granules of densely granulated corticotropinomas were strongly PAS-positive and immunohistochemistry revealed СК7 expression in the cytoplasm of these tumors.

In the corticotropinoma group, the average number cells expressing ACTH was 51.1±17.4% (from 21% to 78%), and the average number cells expressing prolactin was 0.7±0.7% (from 0.1% to 1.8%). Expression of other hormones was not seen. NeuroD1 TF signal was seen in all cases. The average number of NeuroD1 expressing cells was 94.9±4.1% (from 87.8% to 99.7%), and the Ki-67 index was 0.8±0.6% (from 0.1% to 1.7%). When comparing the average numbers of NeuroD1 expressing cells in all adenoma types by immunohistochemistry, statistically significant differences were seen between corticotropinomas and gonadotropinomas (*p*=0.02).

### Mammosomatotropinomas

In this group, there were eight cases of clinically diagnosed somatotropinoma and two cases of prolactinoma. According to magnetic resonance imaging data, the average size of mammosomatotropinomas was 22х16х19 mm. Histological study revealed adenomas with diffuse or sinusoidal structure, consisting of cells with rounded, relatively monomorphic nuclei and distinguished nucleoli. Samples generally showed acidophilic cytoplasm and focal chromophobe cells. All 10 adenomas expressed СК7.

**Figure 5:**
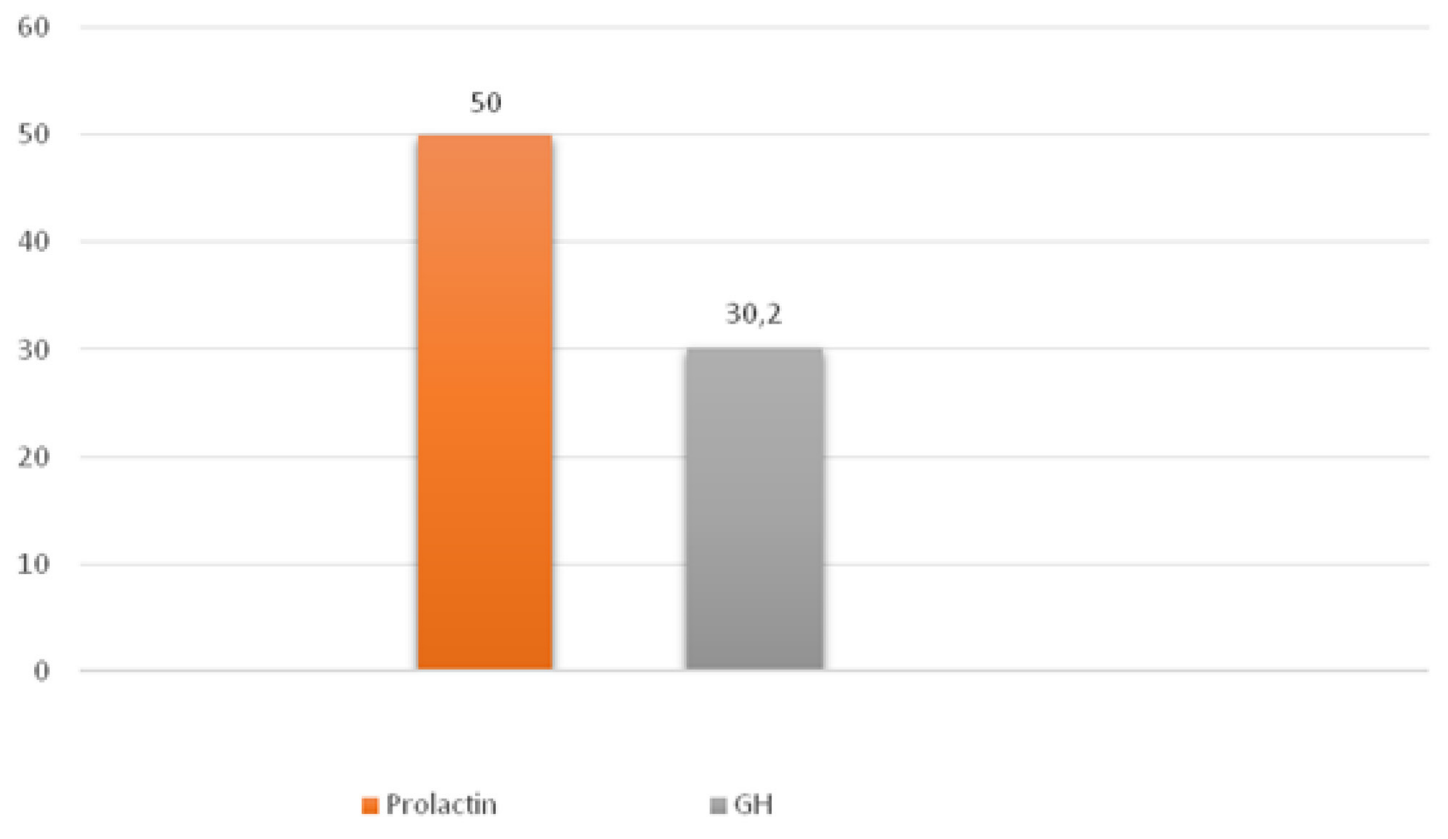
Average hormone expression values in mammosomatotropinomas Numbers indicate the average number of hormone expressing cells (percent).

**Figure 6:**
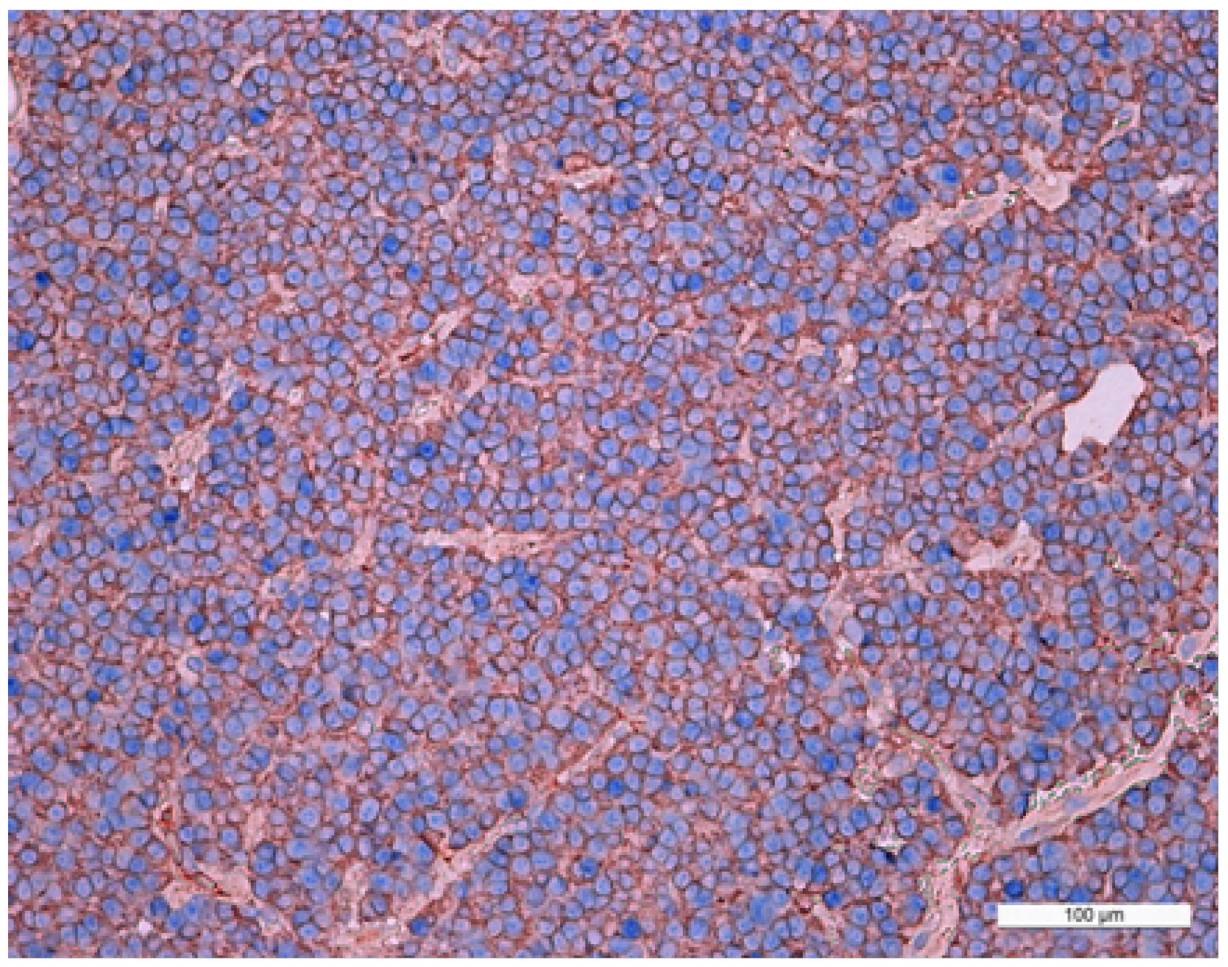
Double stain immunohistochemistry, mammosomatotropinoma, Growth hormone hormone/NeuroD1, ×200 Growth hormone is visualized with red colour, NeuroD1 with blue colour. Same cell co-expression of Growth hormone and NeuroD1 is 100%. Generaliiy, the average number of cells with co-expression (Growth hormone/NeuroD1) in this pituitary adenoma is 99.8%.

In mammosomatotropinomas, the average numbers of cells expressing given markers were: prolactin 50.0±10.3% (33.0-61.2%); GH 30.2±8.8% (18.5-41.1%; Figure 5); and NeuroD1 97.4±2.6% (92.9-99.7%). The Ki-67 index was 1±0.5% (0.3-1.9%). NeuroD1 signal was seen in all samples. Double stain immunohistochemistry revealed PRL and NeuroD1 co-expression in 8% of cells, on average (from 6.5 to 9.3%). GH and NeuroD1 were co-expressed in 99.6% of cells, on average (from 97.6 to 99.8%) (Figure 6).

**Figure 7:**
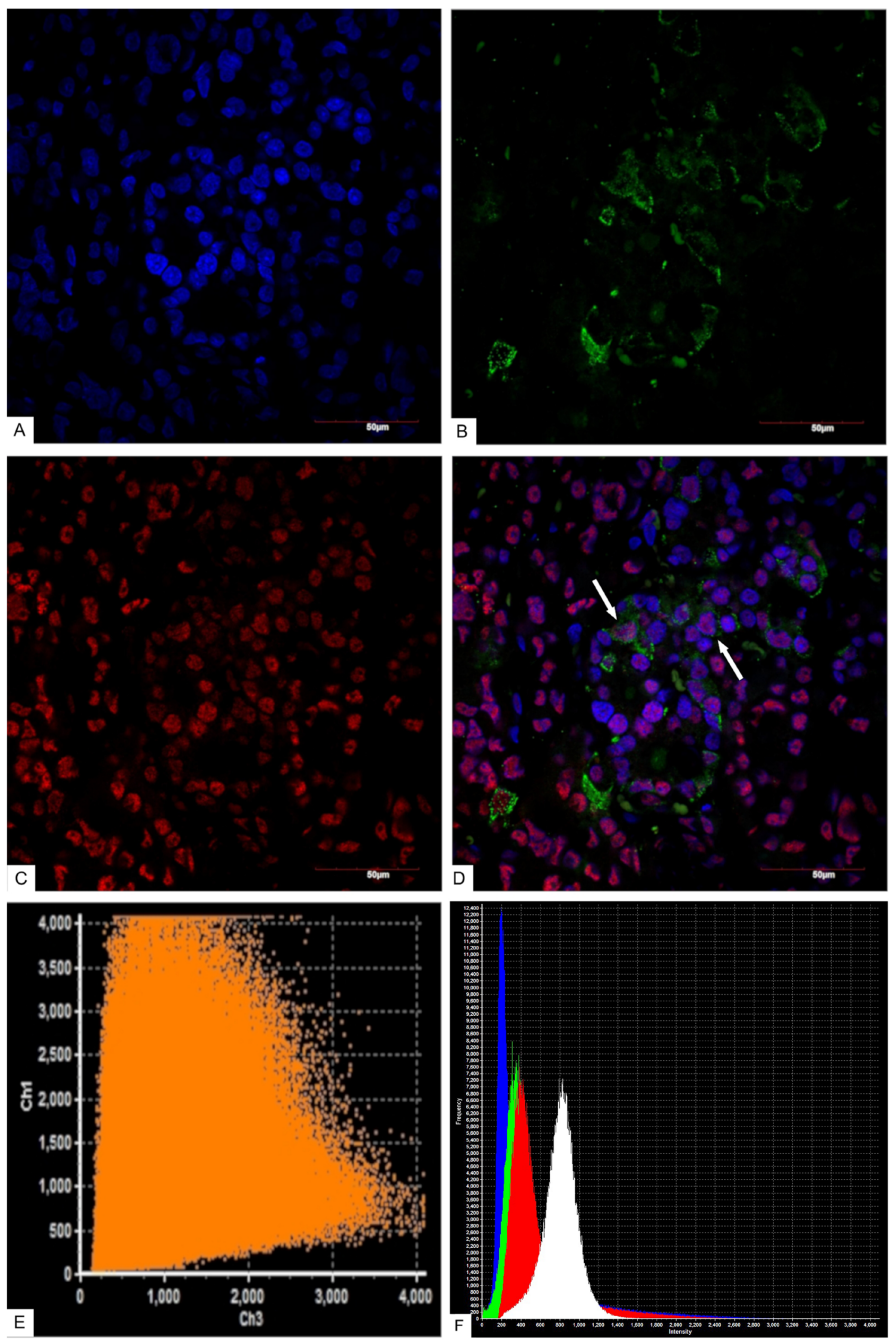

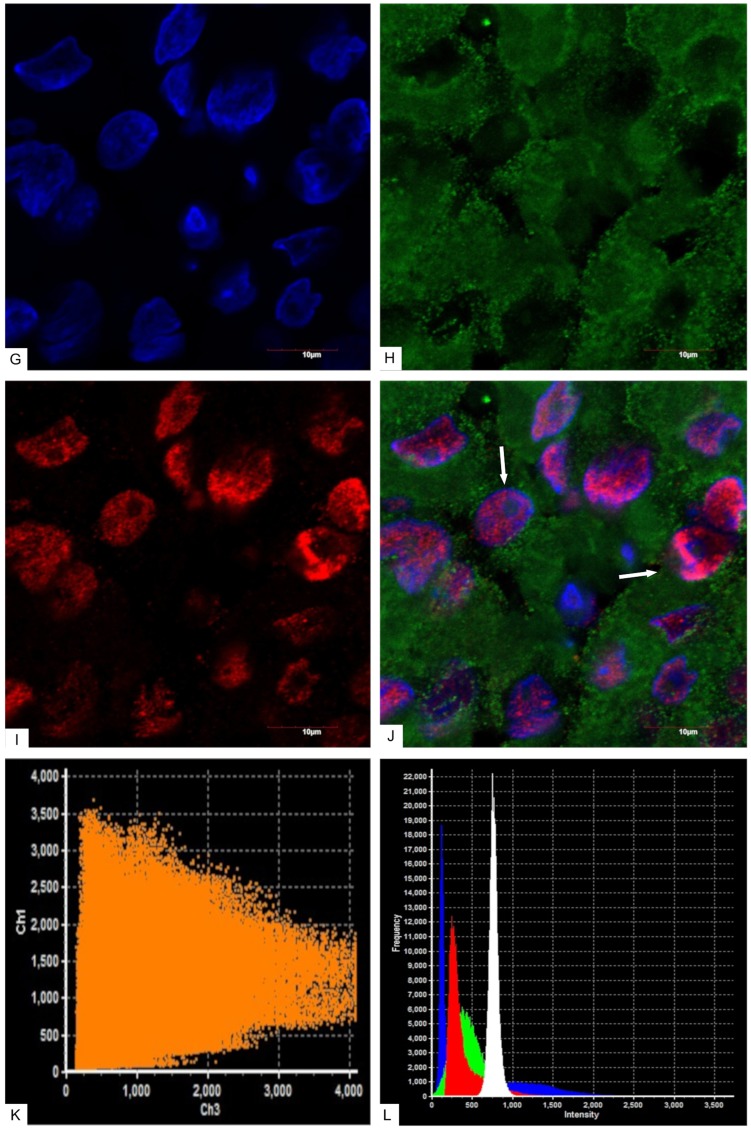
Confocal laser scanning microscopy, mammosomatotropinoma №1 **(A)**: blue fluorescence of cell nuclei (DAPI); **(B)**: green fluorescence of Prolactin; **(C)**: red fluorescence of NeuroD1; **(D)**: overlay image **(A, B, C)**. NeuroD1(pink fluorescence)/Prolactin (green fluorescence) same cell co-expression is seen in 30% of the cells (indicated by arrows); × 600; **(E)**: scatterplot of blue (DAPI, Ch1) and red (Neuro D1, Ch 3) pixel intensities of tumor cell nuclei; **(F)**: intensity histogram of red (Neuro D1), green (Prolactin), and blue (DAPI) fluorescence. White channel: light microscopy; **(G)**: blue fluorescence of cell nuclei (DAPI); **(H)**: green fluorescence of Growth hormone; **(I)**: red fluorescence of NeuroD1; **(J)**: overlay image **(G, H, I)**. NeuroD1(pink fluorescence)/Growth hormone (green fluorescence) same cell co-expression is seen in 77% of the cells (indicated by arrows); × 2400; **(K)**: scatterplot of blue (DAPI, Ch1) and red (Neuro D1, Ch 3) pixel intensities of tumor cell nuclei; **(L)**: intensity histogram of red (Neuro D1), green (Growth hormone), and blue (DAPI) fluorescence. White channel: light microscopy.

According to confocal laser scanning microscopy (CLSM) data, the average NeuroD1 expression coefficient was 75%. In the three mammosomatotropinoma samples (studied by CLSM) the coefficients of prolactin/NeuroD1 co-expression were: 30%; 16%; and 18% (21.3±6.1% average). The GH/NeuroD1 co-expression coefficients were: 77%; 86%; and 90% (84.3±5.4% average). Figure 7 and Supplementary Figure 1 present dual label imaging data regarding the coexpression pairs mentioned above. The intensity of expression (fluorescence) of NeuroD1 was 145-1794 standard units, compared to 36-2135 standard units for DAPI (Figure 7F, 7L and Supplementary Figure 1F, 1L).

**Figure 8:**
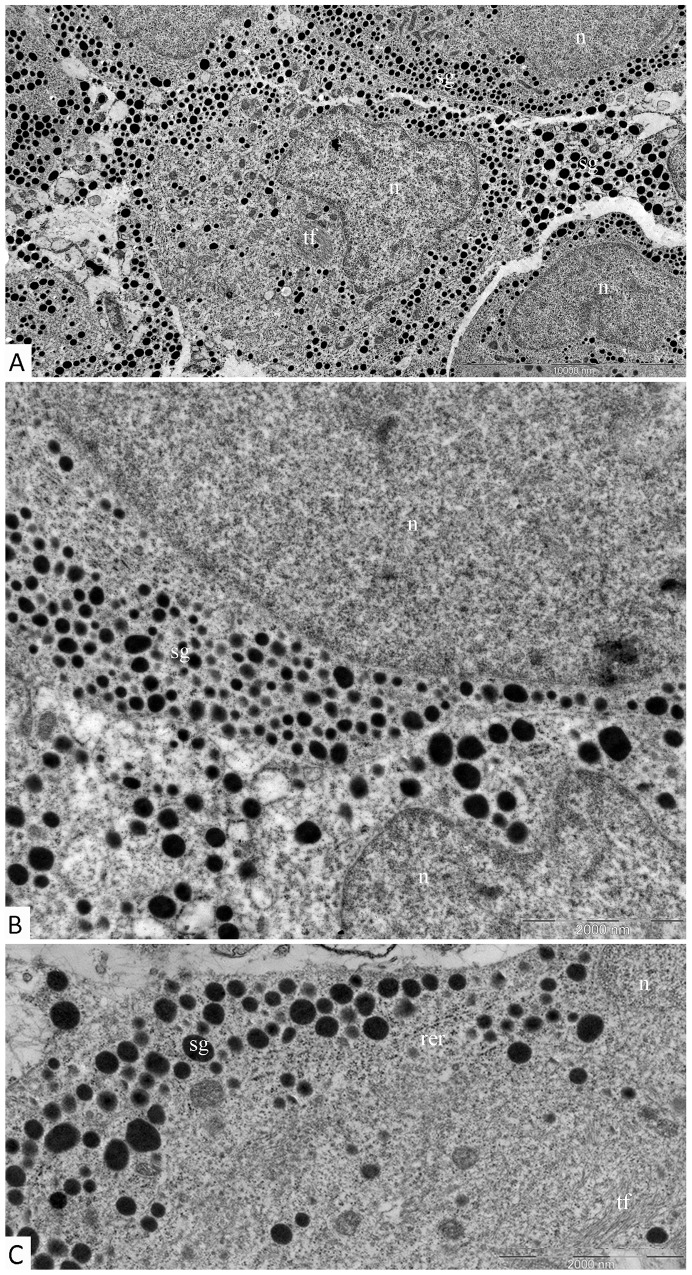
Ultrastructure of mammosomatotropinoma cells **(A)**: general view of mammosomatotropinoma cells; **(B and C)**: various types of secretory granules in the cytoplasm of mammosomatotropinoma cells. Abbreviations: n: nuclei; sg: secretory granules; tf: tonofilaments; rer: rough endoplasmic reticulum.

Electron immunocytochemistry was used to reveal the ultrastructural organization of the mammosomatotropinoma cells and to investigate NeuroD1’s localization therein. It was found that mammosomatotropinoma cell shapes were nearly round, or slightly polygonal, with rounded nuclei, and sometimes having small invaginations (Figure 8A). Cytoplasma were filled with a large number of secretory granules; they varied significantly by size and electron density. Granule diameters ranged from 150 to 600 nm. While large granules had high electron densities, their smaller counterparts ranged from relatively electron-transparent to electron-dense.

**Figure 9:**
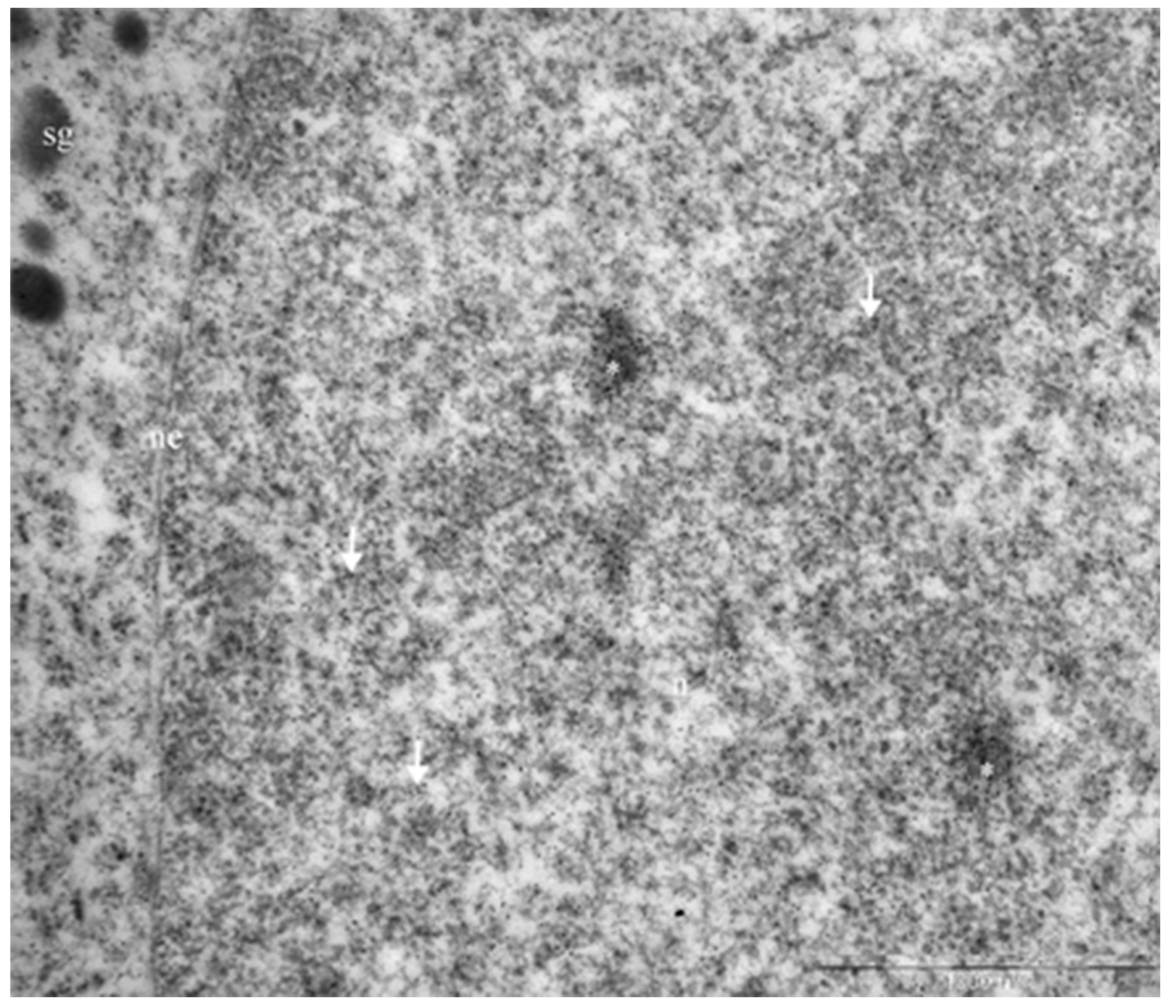
Immunogold labeling of Neuro D1, mammosomatotropinoma cell nucleus Diffuse labeling in the nucleus is seen *(arrows)*, with concentration of the label in 200-300 nm electron-dense nuclear structures *(asterisks)*. Abbreviations: n: nucleus; ne: nuclear envelope; sg: secretory granules.

The distribution of granules in tumor cells varied. We identified cells featuring mainly large (500-600 nm) electron-dense granules (Figure 8B, lower section) and also cells with equally dispersed large and smaller granules (150-400 nm) (Figure 8B, upper section; Figure 8C). Along with secretory granules, specific features seen in mammosomatotropinoma cells included developed rough endoplasmic reticulum (Figure 8C) and an abundance of tonofilaments. In some cases, tonofilaments formed small, rounded inclusions in the perinuclear cytoplasm (Figure 8B), but more often they were organized in separate bundles (Figure 8C). Indirect immunodetection of NeuroD1 by the immunogold method revealed multiple colloidal gold labeling of mammosomatotropinoma cell nuclei; neuroD1 concentration was observed in 200-300 nm electron-dense nuclear structures (Figure 9).

### Prolactinomas

This group included six cases of clinically diagnosed prolactinoma and two cases of nonfunctioning pituitary adenoma. According to magnetic resonance imaging data, the average prolactinoma size was 25х25х24 mm. In this group, densely granulated adenomas were rarely seen. All of the adenomas were chromophobic; they featured solid structure and perivascular pseudorosette formations.

**Figure 10:**
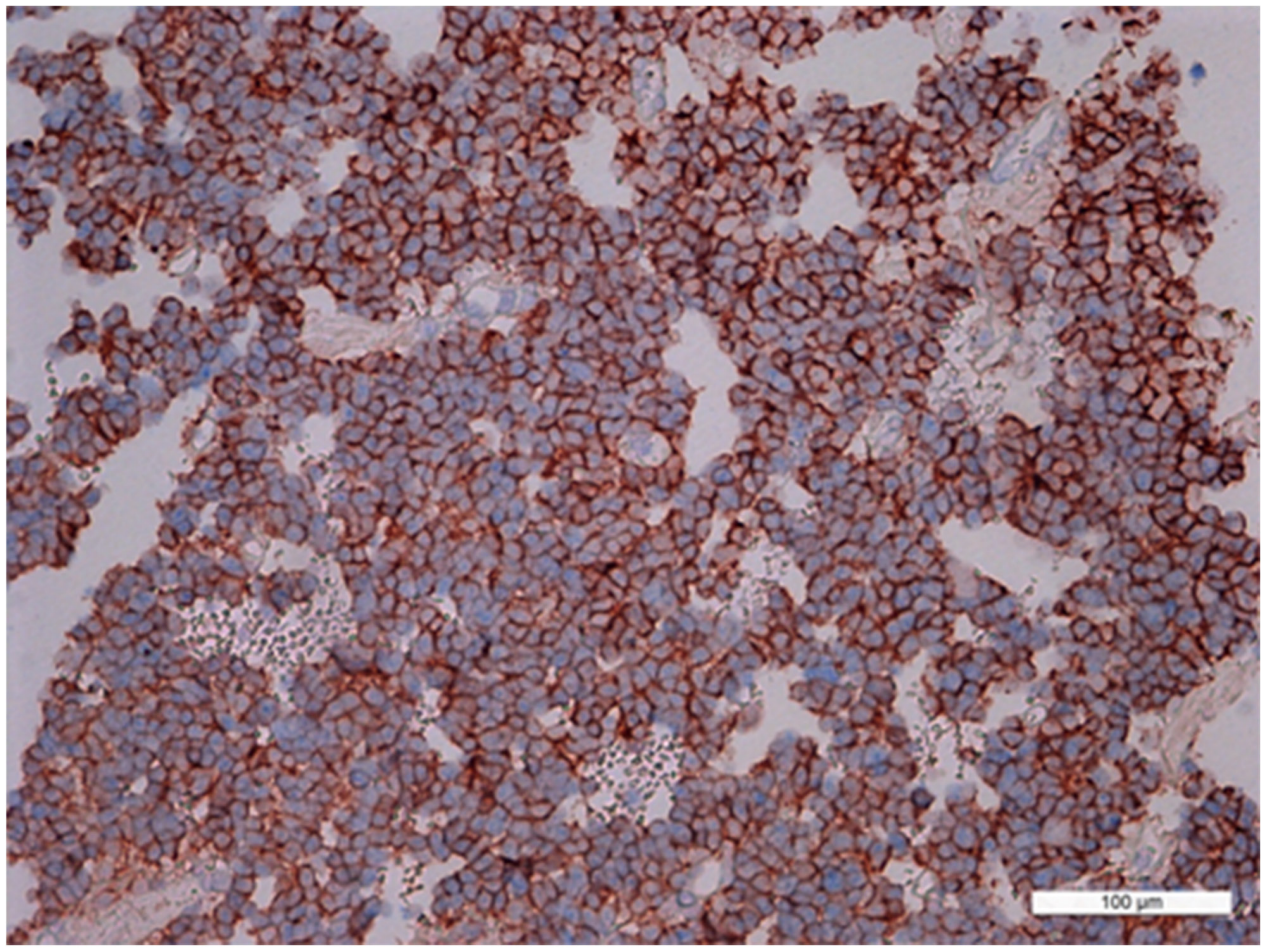
Double stain immunohistochemistry, prolactinoma, Prolactin/NeuroD1, ×200 Prolactin is visualized with red colour, NeuroD1 with blue colour. PRL/NeuroD1 co-expression is seen in 90% of cells.

In prolactinomas, the average numbers of cells expressing given markers were: prolactin 45.8±5.6% (from 40% to 55%); ACTH 0.05±0.08% (0-0.2%); GH 0.03±0.04% (0.04-0.1%); and NeuroD1 98.9±0.6% (98.2-99.3%). The Ki-67 index was 3±2% (0.8-4.6%). NeuroD1 was expressed in the nuclei in all prolactinoma cases. Double stain immunohistochemistry revealed PRL NeuroD1 co-expression in 99.2% of cells, on average (from 88.0 to 100.0%; Figure 10).

### Somatotropinomas

Clinically, somatotropinomas manifested as acromegalia and chiasmal syndrome. The two adenomas were 24×23×22 mm and 28×27×26 mm in size. Histologically, adenoma structure was chromophobe, solid, and with perivascular pseudorosette formations.

**Figure 11:**
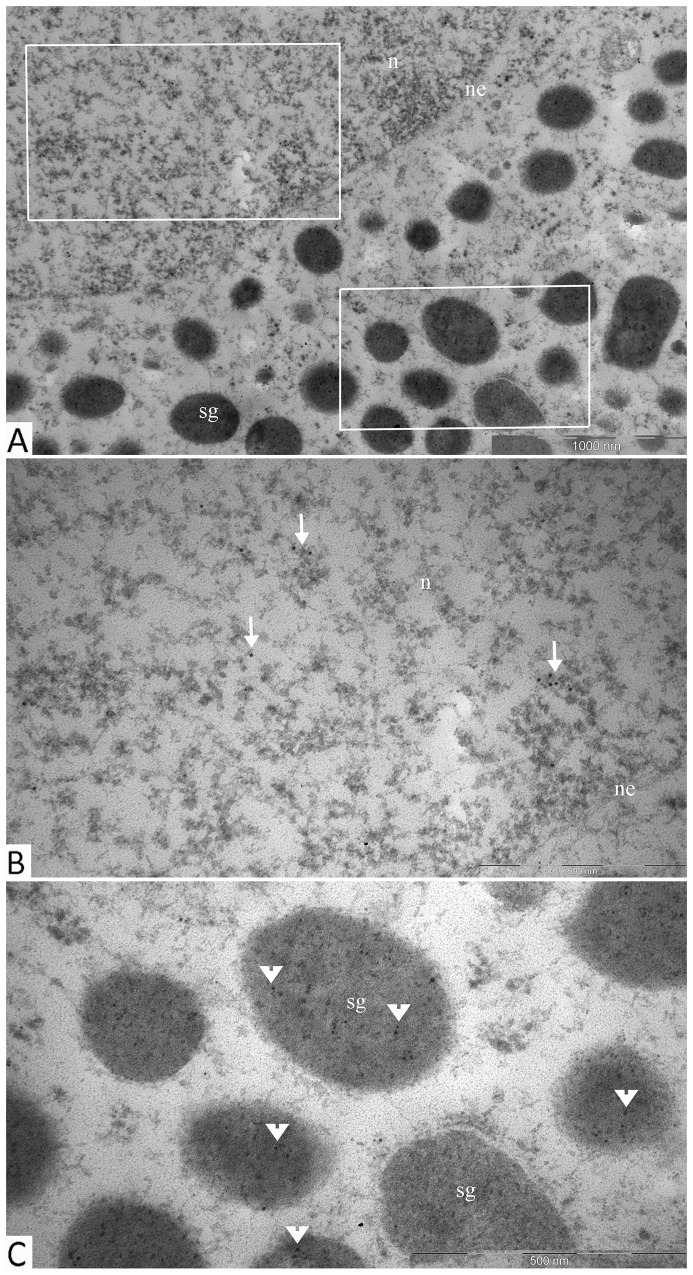
Double immunogold labeling of NeuroD1 and GH, somatotropinoma cell **(A)**: micrograph of part of a single cell with selected areas of its nucleus and cytoplasm containing secretory granules shown in panels *b* and *c*, respectively; **(B)**: the nucleus of the cell is positive for Neuro D1 (10 nm gold particles, *arrows*); **(C)**: secretory granules in the same cell are positive for GH (5 nm gold particles, *arrowheads*). Abbreviations: n: nucleus; ne: nuclear envelope; sg: secretory granules.

In the two specimens, the average numbers of cells expressing markers were: 53.2 and 71.5% for GH; 98.4 and 99.2% (92.9-99.7%) for NeuroD1. The Ki-67 indices were 0.3 and 1.2%. In both cases, electron immunocytochemistry revealed co-expression of NeuroD1 and GH in the same cell. Gold label 10nm in diameter, indicating NeuroD1 detection, was observed in nuclei. Gold label 5nm in diameter, indicating GH detection, was observed in cytoplasmic secretory granules (Figure 11).

### Gonadotropinomas

All 5 gonadotropinomas clinically manifested as non-functioning pituitary adenomas. Patient symptoms in this group were dependent on tumor size. All patients experienced regular headaches, chronic weakness, and tunneling of vision; three of them had olfactory impairment. According to magnetic resonance imaging data, the average gonadotropinoma size was 23х25х27 mm. Gonadotropinomas consisted of elongated cells with monomorphous rounded nuclei, mostly occupying one pole of chromophobic cytoplasm. The tumors had sinusoidal or trabecular structures with focal perivascular pseudorosette formations.

In gonadotropinomas, the average numbers of cells expressing given markers were: LH 24.0±14.5% (10.5-45.8%); FSH 12.8±14.4% (0.2-35.3%); and NeuroD1 99.2±0.4% (98.8-99.8%). The Ki-67 index was 1.1±1% (0.3-2.9%), and NeuroD1 TF signal was seen in all samples. Statistically significant differences in the average number of NeuroD1 expressing cells were registered between gonadotropinomas and null-cell adenomas (*p*=0.004).

### Null-cell adenomas

In this group, non-functioning pituitary adenoma was clinically diagnosed in all of the cases. The clinical presentations of the null-cell adenomas were primarily defined by the size of the tumor. According to magnetic resonance imaging data, the average size of null-cell pituitary adenomas was the largest one among the groups studied and equaled 33х31х31 mm. The six cases with defined adenoma dimensions (according to MRI) comprised 3 macroadenomas (measuring more than 1 cm in one dimension) and 3 giant adenomas (measuring more than 4 cm in one dimension). Histologically, the null-cell adenomas consisted of monomorphous small rounded cells, with acidophilic or chromophobic cytoplasm, forming perivascular pseudorosettes. These tumors had solid or sinusoidal structures. Mitotic cells were not observed in the null-cell adenomas.

Hormone expression was not seen in the null-cell adenomas. Signal corresponding to NeuroD1 expression was seen in all of the cases. The average numbers of cells expressing NeuroD1was 94.1±5.8% (85.1-98.8%). The Ki-67 index was 1.8±1.4 (0.8-3.8%). Thus, the NeuroD1 TF was expressed, on average, in 95.5% of cells in all of the investigated adenoma types.

### Normal adenohypophysis tissue near adenoma boundaries

Normal adenohypophysis areas expressing the majority of hormones, and consisting of expanded adenomeres, were found in 4 adenomas (3 plurihormonal and 1 gonadotropinoma) near tumor boundaries (Table 2). In these fragments, the average numbers of cells expressing given markers were: ACTH 61.8±7.6% (55.3-72.3%); prolactin 46.9±2.5% (44.6-49.6%); GH 28.7±12.0% (15.8-39.5%); LG 31.8±19.9% (4.2-50.8%); FSH 24.1±19.3% (3.1-49.1%); TSH 10.8±21.3% (0.2-42.8%); and NeuroD1 96.1±1.0% (95.2-97.1%).

**Table 2:** Percentage of antigen expressing cells in normal adenohypophysis fragments taken near adenoma boundaries

Antibody	Average number of antigen expressing cells				
	Case number				Group average
	1	2	3	4	
ACTH	62.6%	56.9%	72.3%	55.3%	61.8±7.6%
GH	15.8%	30.8%	39.5%	-	28.7±12.0%
PRL	44.6%	46.6%	49.6%	-	46.9±2.5%
LG	50.8%	32.3%	39.9%	4.2%	31.8±19.9%
FSH	17.1%	26.9%	49.1%	3.1%	24.1±19.3%
TSH	0%	0.2%	0%	42.8%	10.8±21.3%
NeuroD1	95.2%	95.2%	96.8%	97.1%	96.1±1.0%

«-» insufficient material for sectioning.

When the average numbers of NeuroD1 expressing cells were compared in all of the sample groups, including normal adenohypophysis fragments near adenoma boundaries, significant differences were seen between gonadotropinomas and normal adenohypophysis (boundary) fragments (*p*=0.02). Significant differences between the average number of NeuroD1 expressing cells in normal adenohypophysis fragments near adenoma boundaries and in normal pituitary gland were not seen (p=0.8). This fact can be explained by the small number of cases (4).

### Normal adenohypophysis

The pituitary glands investigated were from 9 to 13 mm long, from 6 to 10 mm wide, and from 5 to 8 mm high. Histological analysis included their anterior, intermediate, and posterior lobes. No signs of autolysis, dystrophy, or necrosis of cells were found in any pituitary glands. In the specimens, adenohypophysis comprised 70-75% of the total pituitary gland and consisted of many glandular epithelial cells arranged in cords and clusters which were covered with reticular fibres and capillaries of trabeculae or adenomeres. The trabecular structures of anterior pituitaries were well defined using the Gordon and Sweet staining method for reticular fibres. The histological structures of pituitary glands from patients with cardiovascular diseases and/or chronic heart failure did not differ from those of patients with leukemia or uterine cancer; patients’ cardiovascular status/disease did not have noticeable impacts on the pituitary.

In normal adenohypophysis, the average numbers of cells expressing given markers were: ACTH 44.4±6.6% (34.7-54.6%); prolactin 52.0±3.1% (47.6-56.8%); GH 46.8±8.3% (33.2-59.4%); LG 18.8±2.5% (15.1-22.2%); FSH 51.0±5.0% (44.8-59.7%); TSH 29.8±5.5% (22.5-39.6%); and NeuroD1 67.7±24.8% (14.0-90.2%). There were no statistically significant differences between patients in terms of any of the hormones (above).

**Figure 12:**
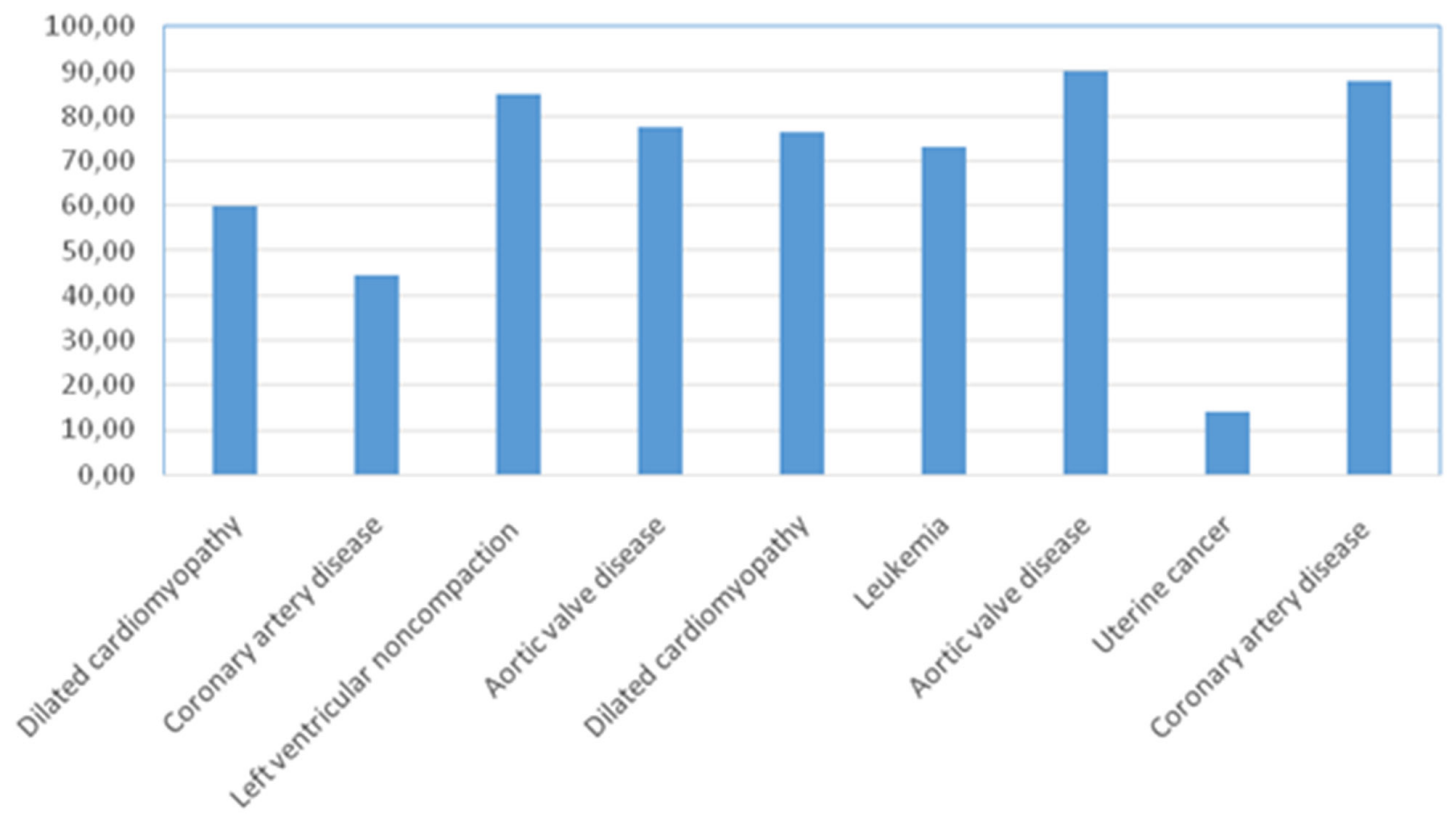
Percentage of Neuro D1 expressing cells in the adenohypophysis of patients without pituitary pathology

**Figure 13:**
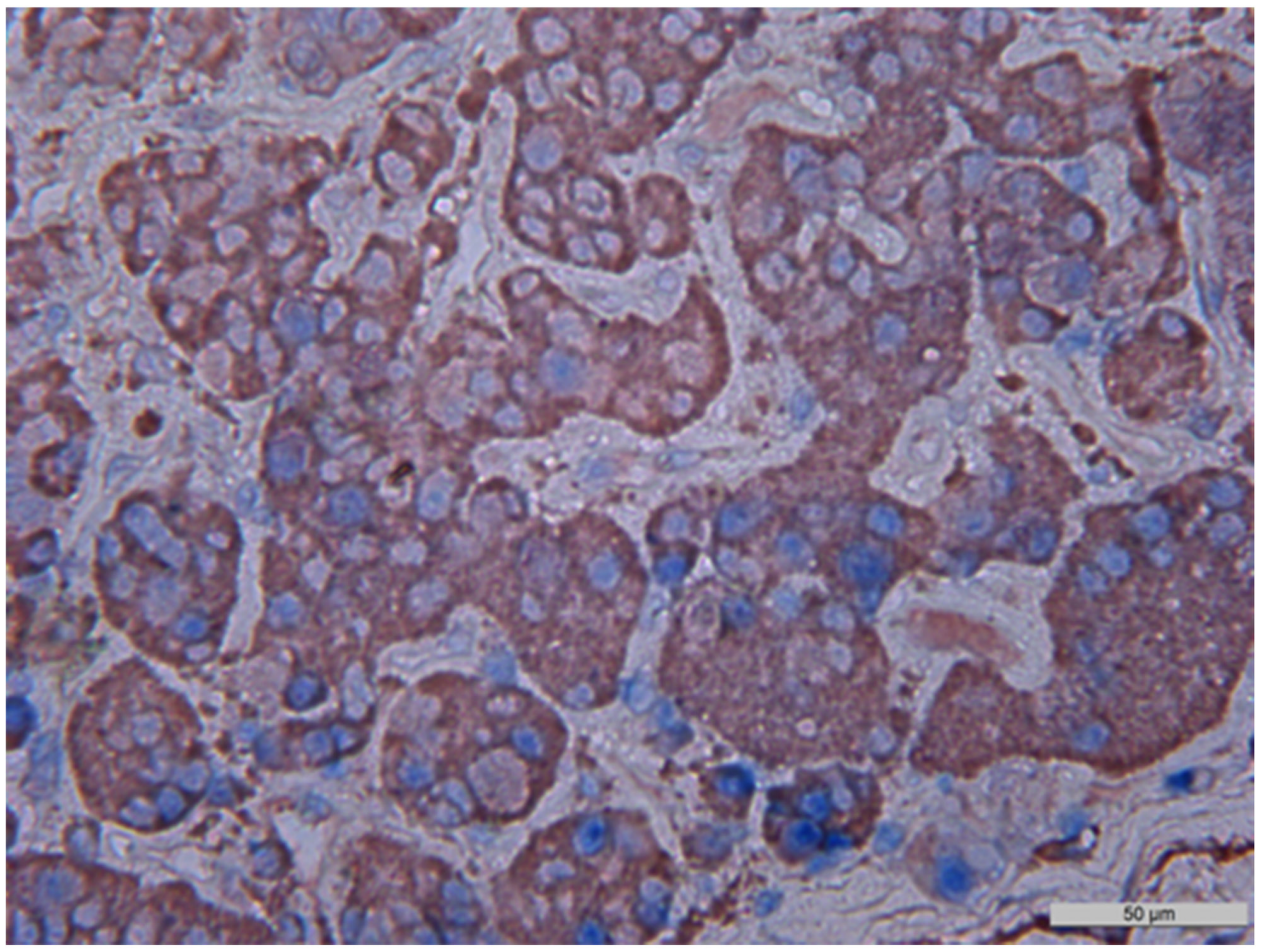
Double stain immunohistochemistry, normal anterior pituitary, Growth hormone/NeuroD1, ×400 Growth hormone is visualized with red colour, NeuroD1 with blue colour. Co-expression (Growth hormone and NeuroD1) is seen in 80% of cells (in this photo; indicated by arrows). GH/NeuroD1 same cell co-expression was 45%, on average in this case.

Table 3 shows that in all of the normal specimens, all of the hormones are expressed in the tissue samples. It is worth mentioning that, unlike adenomas, the average number of NeuroD1 expressing cells in normal anterior pituitary was extremely variable (from 14 to 90.22%; Figure 12). The lowest average number of NeuroD1 expressing cells was in a patient with uterine cancer (case number 8 in Table 3). The expression of hormones in this adenohypophysis specimen did not differ from that in patients with cardiovascular pathology. In a patient with leukemia, the average number of NeuroD1 expressing cells was much higher (case number 6, Table 3). Double stain immunohistochemistry revealed PRL and NeuroD1 co-expression in 50% of cells, on average (from 41 to 58%). Co-expression of GH and NeuroD1 was seen in 45% of cells, on average (from 35 to 58%; Figure 13).

**Table 3:** Percentage of antigen expressing cells in normal adenohypophysis

Antibody	Average number of antigen expressing cells									
	Case number									Group Average
	1	2	3	4	5	6	7	8	9	
ACTH	39.5	52.3	34.7	41.9	42.4	54.6	38.7	48.8	46.9	44.4±6.6%
GH	33.2	53.7	36.9	49.1	42.4	45.2	59.4	49.7	51.4	46.8±8.3%
PRL	49.3	47.6	53.8	52.9	56.8	48.3	52.7	55.2	51.5	52.0±3.1%
LH	22.1	16.9	17.8	20.5	15.1	21.8	16.3	20.7	18.4	18.8±2.5%
FSH	48.3	45.2	59.7	51.4	49.5	50.3	52.9	57.1	44.8	51.0±5.0%
TSH	39.6	30.3	35.2	25.7	22.5	24.8	27.4	29.1	33.8	29.8±5.5%
NeuroD1	59.94	44.46	85.03	77.73	76.65	73.12	90.22	14.0	87.96	67.7±24.8%

**Figure 14:**
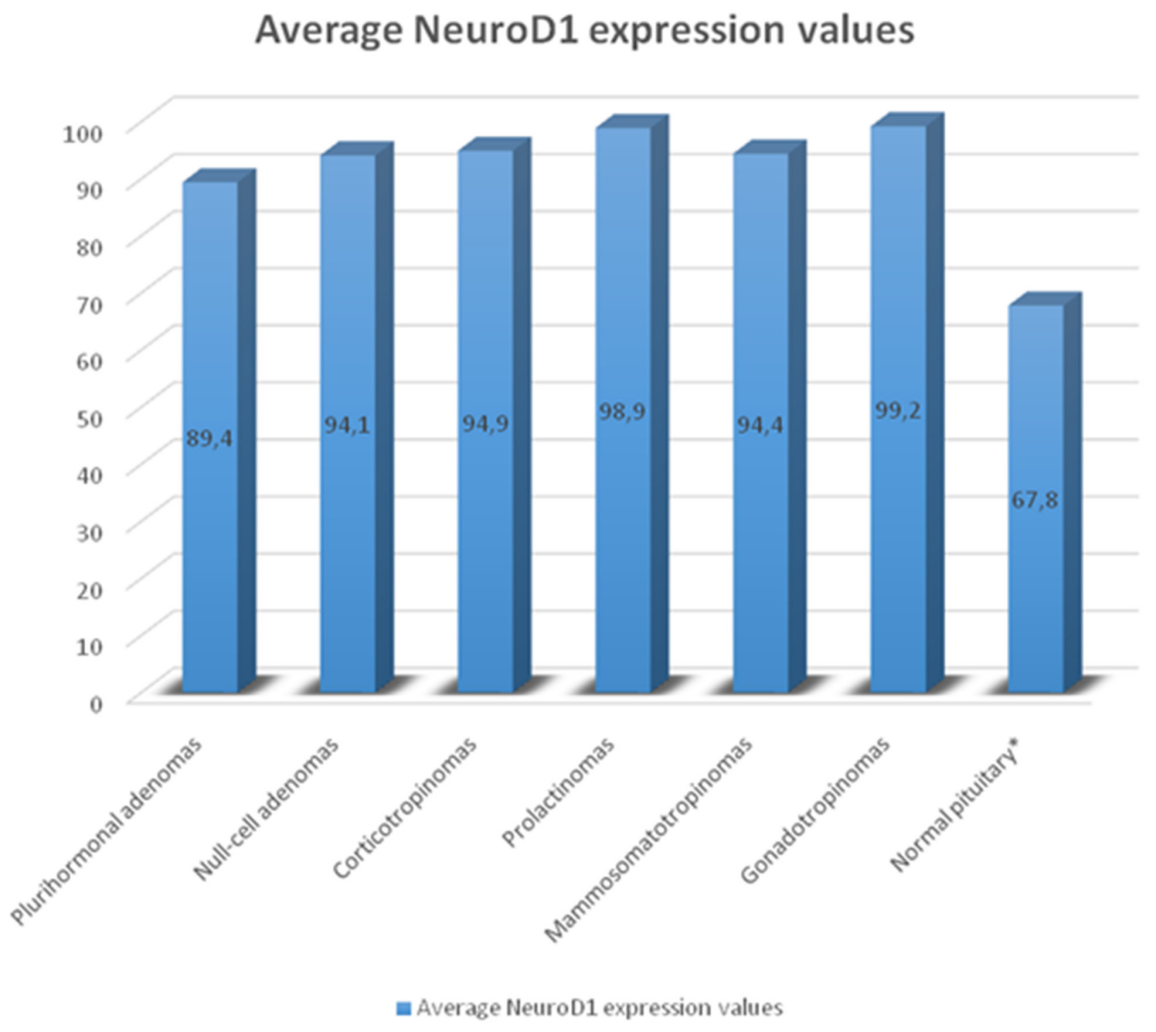
Average number of NeuroD1 expressing cells in normal adenohypophysis specimens and adenomas Significant differences in Neuro D1 values between the normal adenohypophysis group and adenoma groups marked with an asterisk.

**Figure 15:**
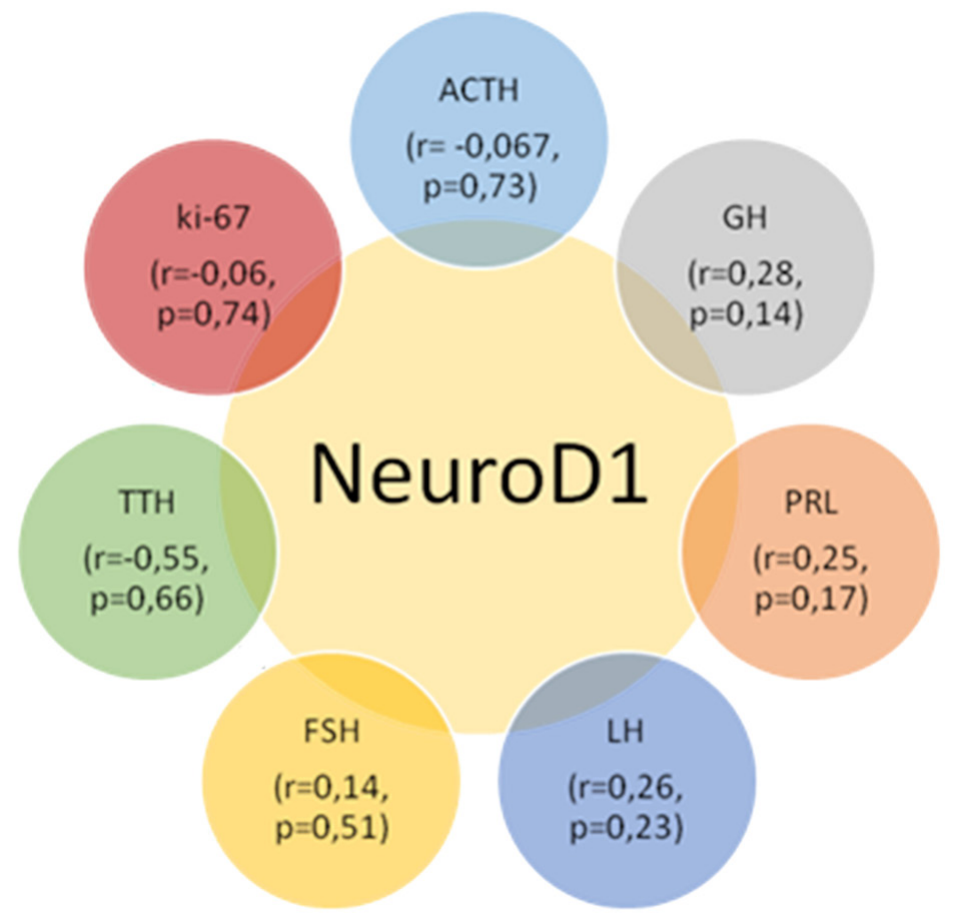
Correlation values between the average numbers of cells with hormone, Neuro D1 and Ki-67 expression in the pituitary gland; p>0.05

**Figure 16:**
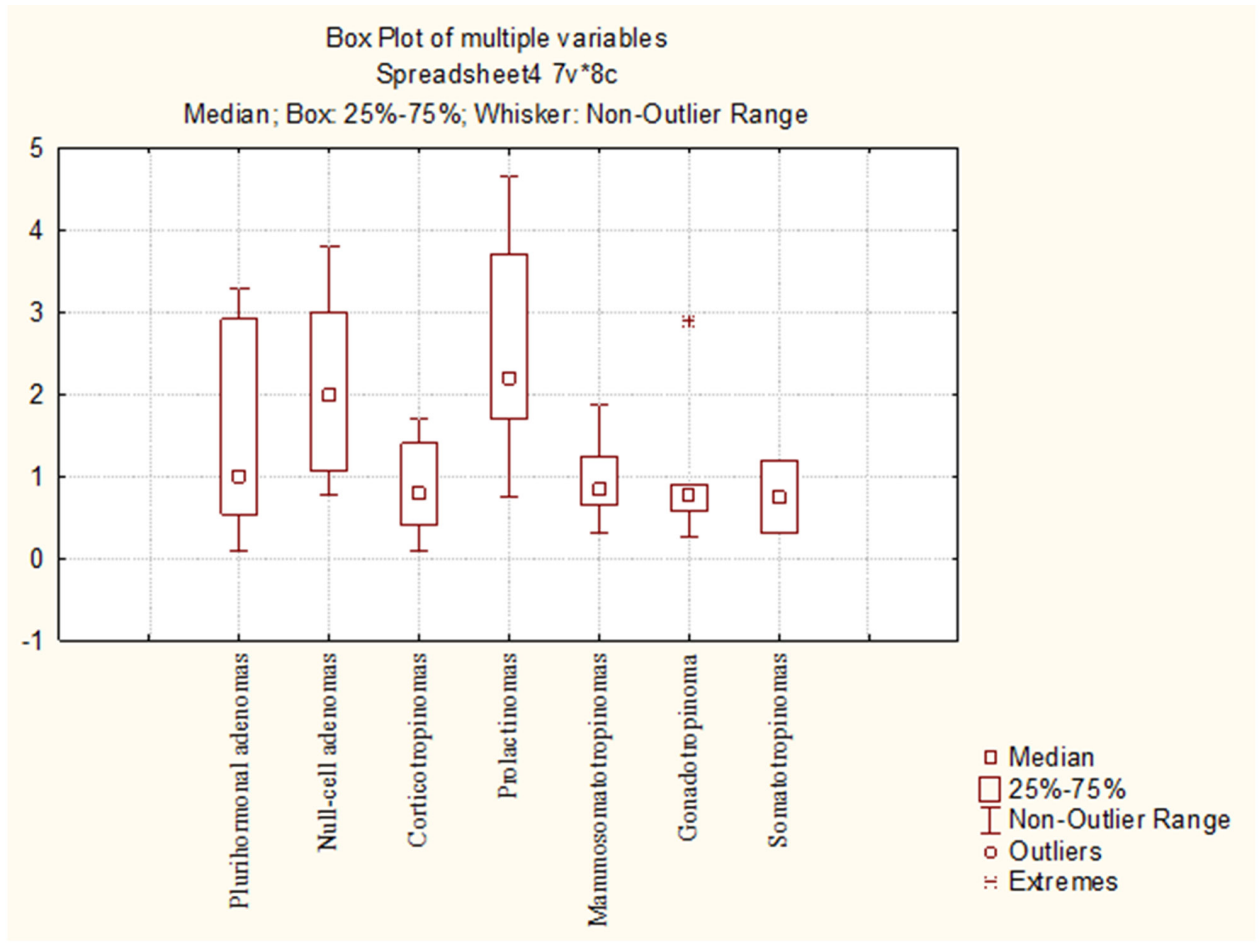
Mean Ki-67 values in different pituitary adenoma groups

The average numbers of NeuroD1 expressing cells in normal adenohypophysis specimens were significantly lower than in the adenomas overall (*p*=0.006; Figure 14). In addition, significant differences were seen between the average number of pituicytes expressing NeuroD1 in normal anterior pituitary and in gonadotropinomas (*p*=0.037), mammosomatotropinomas (*p*=0.019), corticotropinomas (*p*=0.019), or null-cell adenomas (*p*=0.019). Correlation analysis did not show any significant relationships between the expression levels of NeuroD1, hormones, or Ki-67 in the pituitary gland (p>0.05; Figure 15). There were no statistically significant differences between different types of adenomas in terms of mean Ki-67 values (Figure 16).

## DISCUSSION

In this study, we performed immunodetection of NeuroD1 in various types of pituitary adenomas. Using immunohistochemistry, we have shown that NeuroD1 is expressed not only incorticotropinomas, but in fact in all study samples, including plurihormonal and null-cell pituitary adenomas, prolactinomas, somatotropinomas, mammosomatotropinomas, and gonadotropinomas. In fact, the expressing cell average was 96% in the entire study. A number of methods were used to clarify NeuroD1’s expression status. In prolactinomas, expression was confirmed by double stain immunohistochemistry. In mammosomatotropinomas, double stain immunohistochemistry and confocal laser scanning microscopy were used. Electron immunocytochemistry was used to evaluate somatotropinomas.

The data we have acquired are in contrast with a series of pituitary adenoma studies and concepts of pituitary gland embryogenesis [18, 19, 20]. However, NeuroD1 expression has, in fact, been detected in the null-cell pituitary adenomas [21]. NeuroD1 expression levels were notably higher in “silent” corticotropinomas [22]. Ferretti et al. [23] found NeuroD1 in all types of pituitary adenomas, but not in all cases. Takiguchi et al. revealed significant expression of NeuroD1 and Pit-1 mRNA in plurihormonal adenomas secreting ACTH and GH [24]. NeuroD1 protein has been detected in the nucleus of non-tumorous anterior pituitary cells, with localization mainly in corticotroph cells which process proopiomelanocortin into ACTH [25].

On the one hand, we have also detected NeuroD1 in normal pituitary cells; on the other hand, the results of our previous investigation proved that normal pituitary cells are plurihormonal [26]. The present work shows that this TF is not only expressed in ACTH-expressing cells. The same results were obtained with normal adenohypophysis taken near adenoma boundaries. Earlier, NeuroD1’s role was understood to be solely connected with the process of embryonic corticotroph formation [27]. However, the data we present suggest that NeuroD1 likely has additional roles. Its widespread expression, in various tissue types, supports this idea.

It should be noted that, like our study, relatively higher NeuroD1 expression levels in adenomas, compared to normal pituitary, have been demonstrated by Fratticci et al. [28]. Such differences in NeuroD1 expression levels in adenomas versus in normal glands may point to a significant role in tumorigenesis. Interestingly, in terms of the average numbers of pituicytes expressing the transcription factor, normal pituitary specimens taken near adenoma boundaries were comparable to adenomas and were much higher than in the normal adenohypophysis (control group). However, the differences we observed between the average number of NeuroD1 expressing cells in normal adenohypophysis fragments near adenoma boundaries and in normal pituitary gland did not reach the statistical significance. This fact can be explained by the small number of cases (4) which were available for the study. This finding implies that NeuroD1 may play key roles in adenoma tumor development or recurrence.

NeuroD1 is expressed not only in the pituitary gland, but also in pancreatic precursor cells, which subsequently differentiate into pancreatic endocrine cells [29, 30]. In addition, NeuroD1 has been detected in the neuroectoderm cells [31]. Moreover, the transcription factor participates in the activation of various genes in adult endocrine, enteroendocrine and neuroendocrine cells; these cells may secrete insulin-1 [32, 33], glucokinase [34], secretin [35], and/or inositol 1,4,5-trisphosphate receptor (IP3R1) [36]. NeuroD1 also plays an important role in the differentiation, morphogenesis, and normal functioning of central nervous system cells [37].

Tani et al. [38] found that NeuroD1 levels were comparable between carcinoid tumors causing ectopic ACTH syndrome and pituitary tumors causing Cushing’s disease. This fact indicates that NeuroD1’s role in pathogenesis may not be limited to only pituitary tumors; it may play roles in other neuroendocrine tumors as well. The wide range of functions described in the literature for this TF indicate that it is of particular significance. NeuroD1’s consistently high expression levels in all types of pituitary adenomas make it an attractive potential target for new drugs designed to reduce its expression. Ideally, such a NeuroD1-targeting drug could be used for the treatment of aggressive or recurrent neuroendocrine tumor cases. The success of such a drug would be predicated on its ability to reduce NeuroD1 expression to levels near those seen in the normal pituitary gland.

Given the fact that NeuroD1 levels are significantly higher in pituitary adenomas than in normal pituitary gland, this protein may be a prognostic factor. In this study, we did not see significant correlation between NeuroD1 and Ki-67 expression in tumor cells. In our opinion, this can be explained by the fact that Ki-67 is neither an ideal indicator nor a sole predictor for pituitary adenoma. This view is supported by de Aguiar et al. [39]. Salehi et al. [40] suggest that inconsistencies in data regarding Ki-67’s role in tumors may be due to differences in the ways in which different research groups study tumors and their manifestations. In particular, the research methodologies used for tumor study are have not been standardized. Different authors use varying criteria of tumor invasion and recurrence. Zakir et al. [41] suggest using several prognostics markers simultaneously, not simply by Ki-67 alone. Such a multiplex approach seems appropriate in light of the complexity of the situation.

## MATERIALS AND METHODS

### Clinical samples

48 pituitary adenomas and 9 normal pituitary glands were studied. Adenomas were removed by endoscopictranssphenoidal surgery and they represented a variety of tumor types (7 plurihormonal adenomas, 8 corticotropinomas, 10 mammosomatotropinomas, 8 prolactinomas, 2 somatotropinomas, 5 gonadotropinomas, and 8 null-cell adenomas). Normal pituitary glands were obtained from patients who died from cardiovascular or oncological diseases. Pituitary glands were taken within 4 hours after death. None of the patients were treated with prolonged corticosteroids, antineoplastic drugs, or other treatments that affect endocrine status or function of the pituitary. A patient with leukemia (acute myeloblastic leukemia) received induction chemotherapy with cytarabine and daunorubicin (“7 + 3”) and did not receive hormone therapy. The patient did not have neuroleukemia; clinically, her pituitary function was not impaired. Patients with pituitary adenomas were aged from 14 to 74 years (50±15 years on average), and the group comprised 32 women and 16 men. The control group (normal pituitary) was comprised of 5 women and 4 men; the mean age was 58±11.7 years, and the ages ranged from 33 to 73 years old. Histological study included hematoxylin and eosin staining, PAS-reaction, and the Gordon-Sweet silver staining methods. Table 4 summarizes data on the clinical characteristics of the patients included in the study, as well as the specific methods used to study samples.

**Table 4:** Patient clinical characteristics and methodological details

№	Sex	Age	Patient pituitary adenoma type, according to IHC or comorbid conditions in normal pituitary donors (autopsy) with duration	Type of pituitary adenoma, according to clinical data or corticosteroid, antineoplasic drug history (autopsies)	Proximal cause of death and duration of that cause	Adenoma or normal pituitary dimensions (in mm, by MRI or autopsy)	Methods applied
1	F	43	Plurihormonal adenoma	Corticotropinoma	-	22×19×15	H, IHC
2	F	69	Plurihormonal adenoma	Somatotropinoma	-	5×6×8	H, IHC
3	F	40	Plurihormonal adenoma	Hormone-inactive adenoma	-	12×10×11	H, IHC
4	F	40	Plurihormonal adenoma	Hormone-inactive adenoma	-	9×6×7	H, IHC
5	M	30	Plurihormonal adenoma	Prolactinoma	-	25×28×18	H, IHC
6	F	35	Plurihormonal adenoma	Corticotropinoma	-	12×12×6	H, IHC, DSIHC
7	M	41	Plurihormonal adenoma	Somatotropinoma	-	34×42×28	H, IHC, DSIHC
8	M	63	Corticotropinoma	Corticotropinoma	-	28×23×20	H, IHC
9	M	37	Corticotropinoma	Prolactinoma	-	48×45×40	H, IHC
10	F	61	Corticotropinoma	Corticotropinoma	-	13×12×15	H, IHC
11	F	35	Corticotropinoma	Corticotropinoma	-	8×7×5,5	H, IHC
12	F	67	Corticotropinoma	Corticotropinoma	-	13×10×9	H, IHC
13	F	58	Corticotropinoma	Corticotropinoma	-	8×6×6	H, IHC
14	F	48	Corticotropinoma	Corticotropinoma	-	9×7×6	H, IHC
15	F	31	Corticotropinoma	Corticotropinoma	-	11,5×6×14	H, IHC
16	M	48	Mammosomatotropinoma	Somatotropinoma	-	16×14×9	H, IHC
17	M	42	Mammosomatotropinoma	Somatotropinoma	-	22×15×10	H, IHC
18	M	30	Mammosomatotropinoma	Somatotropinoma	-	23×19×22	H, IHC, DSIHC
19	M	63	Mammosomatotropinoma	Somatotropinoma	-	12×18×19	H, IHC, DSIHC
20	F	57	Mammosomatotropinoma	Prolactinoma	-	15×18×14	H, IHC
21	M	26	Mammosomatotropinoma	Somatotropinoma	-	34×26×39	H, IHC, CLSM
22	F	64	Mammosomatotropinoma	Somatotropinoma	-	9×13×14	H, IHC, CLSM
23	F	58	Mammosomatotropinoma	Somatotropinoma	-	9×2×7	H, IHC, CLSM
24	F	39	Mammosomatotropinoma	Somatotropinoma	-	18×14×13	H, IHC, EICC
25	F	20	Mammosomatotropinoma	Prolactinoma	-	22×21×19	H, IHC, EICC
26	M	68	Prolactinoma	Prolactinoma	-	30×33×35	H, IHC
27	F	40	Prolactinoma	Prolactinoma	-	19×16×12	H, IHC
28	F	53	Prolactinoma	Prolactinoma	-	16×17×10	H, IHC
29	F	51	Prolactinoma	Prolactinoma	-	27×37×32	H, IHC
30	F	61	Prolactinoma	Hormone-inactive adenoma	-	22×27×19	H, IHC
31	F	40	Prolactinoma	Prolactinoma	-	9×6×7	H, IHC
32	M	27	Prolactinoma	Prolactinoma	-	17×25×26	H, IHC, DSIHC
33	M	56	Prolactinoma	Hormone-inactive adenoma	-	25×29×20	H, IHC, DSIHC
34	F	34	Somatotropinoma	Somatotropinoma	-	7×9×11	H, IHC, EICC
35	F	46	Somatotropinoma	Somatotropinoma	-	16×13×19	H, IHC, EICC
36	F	62	Gonadotropinoma	Hormone – inactive adenoma	-	19×24×17	H, IHC
37	F	71	Gonadotropinoma	Hormone – inactive adenoma	-	24×28×34	H, IHC
38	F	65	Gonadotropinoma	>Hormone – inactive adenoma	-	21×22×20	H, IHC
39	M	62	Gonadotropinoma	Hormone – inactive adenoma	-	14×19×9	H, IHC
40	F	63	Gonadotropinoma	Hormone – inactive adenoma	-	27×18×24	H, IHC
41	F	54	Null-cell adenoma	Hormone – inactive adenoma	-	21×25×26	H, IHC
42	F	66	Null-cell adenoma	Hormone – inactive adenoma	-	15×6×9	H, IHC
43	F	44	Null-cell adenoma	Hormone – inactive adenoma	-	20×17×19	H, IHC
44	M	74	Null-cell adenoma	Hormone – inactive adenoma	-	44×47×43	H, IHC
45	M	14	Null-cell adenoma	Hormone – inactive adenoma	-	38×41×59	H, IHC
46	M	69	Null-cell adenoma	Hormone – inactive adenoma	-	30×24×27	H, IHC
47	F	67	Null-cell adenoma	Hormone – inactive adenoma	-	19×12×15	H, IHC
48	F	70	Null-cell adenoma	Hormone – inactive adenoma	-	53×50×37	H, IHC
49	M	65	Normal pituitary (autopsy) Dilated cardiomyopathy (65 years)	No use of corticosteroid or anticancer drugs	Heart failure (3 years)	11х10×7	H, IHC, DSIHC
50	M	68	Normal pituitary (autopsy) Coronary artery disease (5 years)	No use of corticosteroid or anticancer drugs	Myocardial infarction (3 days)	11х8×7	H, IHC
51	F	28	Normal pituitary (autopsy) Left ventricular noncompaction (28 years)	No use of corticosteroid or anticancer drugs	Pulmonary embolism (<1 day)	9х6×5	H, IHC
52	M	41	Normal pituitary (autopsy) Aortic valve disease (41 years)	No use of corticosteroid or anticancer drugs	Heart failure (1 year)	13х6×6	H, IHC
53	M	55	Normal pituitary (autopsy)Dilated cardiomyopathy (55 years)	No use of corticosteroid or anticancer drugs	Heart failure (4.5years)	12х7×7	H, IHC
54	F	19	Normal pituitary (autopsy)Leukemia (11 months)	cytarabine and daunorubicin; without corticosteroid use	Pneumonia (7 days)	9х8×6	H, IHC
55	M	63	Normal pituitary (autopsy) Aortic valve disease (10 years)	No use of corticosteroid or anticancer drugs	Heart failure (1.5 year)	10х8×7	H, IHC
56	F	48	Normal pituitary (autopsy) Uterine cancer (radical hysterectomy and radiotherapy 5 years prior)	No use of corticosteroid or anticancer drugs	Cancer intoxication (1 year)	9х7×6	H, IHC
57	M	69	Normal pituitary (autopsy) Coronary artery disease (9 years)	No use of corticosteroid or anticancer drugs	Myocardial infarction (< 1 day)	11×9×8	H, IHC

H: histology; IHC: immunohistochemistry; DSIHC: double stain immunohistochemistry; CLSM: confocal laser scanning microscopy; EICC: electron immunocytochemistry; MRI: magnetic resonance imaging.

### Antibodies

For immunohistochemical staining, confocal microscopy, and electron immunocytochemistry, the following primary antibodies were used:

mouse monoclonal ACTH antibody, diluted 1:500 (clone AH26, Diagnostic BioSystems, Netherlands)

rabbit polyclonal TSH antibody, RTU (Cell Marque, USA)

mouse monoclonal FSH antibody, diluted 1:100 (clone С10, DAKO, Denmark)

mouse monoclonal LH antibody, diluted 1:500 (clone С93, DAKO, Denmark)

rabbitpolyclonal GH antibody, diluted 1:100 (BioGenex, USA)

rabbit polyclonal PRL antibody, diluted 1:700 (DAKO, Denmark)

mouse monoclonal NeuroD1 antibody, diluted 1:1000 (clone ab60704, Abcam, United Kingdom)

mouse monoclonal Ki-67antibody, diluted 1:200 (clone MIB-1, DAKOCytomation, Denmark)

mouse monoclonal CK7antibody, diluted 1:300 (clone OV-TL 12/30, DAKO, Denmark)

The following secondary antibodies/reagents were used for immunohistochemical staining:

mouse EnVision™+ System, Peroxidase (DAKO, Denmark)

rabbit EnVision™+ System, Peroxidase (DAKO, Denmark)

MultiVision Polymer Cocktail (Thermo Scientific, UK)

The following secondary antibodies were used for confocal microscopy:

Alexa Fluor 647 goat anti-Mouse, diluted 1:100 (Abcam, UK)

Alexa Fluor 488 goat anti-Rabbit, diluted 1:100 (Abcam, UK)

The following secondary antibodies were used for electron immunocytochemistry:

goat-anti mouse antibody conjugated to 10nm colloidal gold, diluted 1:100 (Sigma-Aldrich, US)

goat-anti rabbit antibody conjugated to 5nm colloidal gold, diluted 1:100 (Sigma-Aldrich, US)

### Immunohistochemistry

For all samples, immunohistochemical study of paraffin sections, using peroxidase-based detection, was performed by one step primary staining with antibodies to NeuroD1, growth hormone (GH), prolactin (PRL), thyroid-stimulating hormone (TSH), adrenocorticotropic hormone (ACTH), luteinizing hormone (LH), follicle-stimulating hormone (FSH)*,* Ki-67, and CK7. Additionally, immunohistochemical double-staining (GH/NeuroD1 and PRL/NeuroD1 cocktails) was used in 2 cases of plurihormonal adenoma, in 2 prolactinomas, in 2 mammosomatotropinomas, and in 1 normal pituitary sample. In order to verify that vendor anti-Neuro D1 antibodies are specific to the D1 isoform of the transcription factor, we performed immunohistochemical staining (anti-Neuro D1) of skeletal muscle sections as a negative control; the muscle section stainings were completely negative (Supplementary Figure 2). The complete immunohistohemical staining method, as used here, is provided in the Supplementary Materials.

### Confocal laser scanning microscopy

In 4 mammosomatotropinomas, confocal laser scanning microscopy (Olympus FV1000D, Japan) was performed using the same primary antibodies (GH/NeuroD1 and PRL/NeuroD1 cocktail). Alexa Fluor 488 goat anti-rabbit and Alexa Fluor 647 goat anti-mouse (Abcam, UK) were used as secondary antibodies. Nuclei were stained with DAPI (appliChem). Details of the confocal laser scanning microscopy method are given in the Supplementary Materials.

### Electron immunocytochemistry

Electron immunocytochemistry was performed as a post-embedding procedure on ultrathin sections of LR White-embedded specimens, with indirect immunolabelling of protein of interest. NeuroD1 immunodetection by electron immunocytochemistry was performed on 2 mammosomatotropinomas, and electron immunocytochemistry with double detection (NeuroD1 and GH) was performed on 2 somatotropinomas (Table 4). The complete procedure is provided in the Supplementary Materials.

### Morphometry and statistics

Morphometric analysis was performed using an automated image analyzer (Image Scope Color M, Russia). In order to analyze the relative quantities of cells expressing select antigens, 10 high power fields (400x magnification) were evaluated per specimen. For all of the hormones and NeuroD1, percentages of the average number of expressing cells, in relation to overall pituicytes, were separately calculated. In addition, percentages of the average number of of cells co-expressing two markers, in relation to overall pituicytes, were calculated, as follows: (GH+NeuroD1)/total or (PRL+NeuroD1)/total.

Statistical analysis of the acquired data was done using Statistica v.10 software (StatSoft, Russia). For normal distributions, the significance of differences in quantitative characteristics was interpreted using the Student’s *t*-test. For other types of distribution, we used non-parametric methods of analysis, namely the Mann-Whitney test for independent samples and the Wilcoxon test. Differences between groups were defined as significant when *p*<0.05.

In order to evaluate the correlation of two variables, we applied Spearman rank correlation analysis. Correlation coefficient (r) interpretation: r <0.3: weak association; r=0.3-0.5: moderate; r= 0.5-0.7: significant; r=0.7-0.9: strong; and r>0.9: very strong. Correlation was considered as positive if r>0 and negative if r<0.

## CONCLUSIONS

Data from a number of methods (immunohistochemistry, confocal microscopy, and double label electron immunocytochemistry) suggest that NeuroD1 plays a key role in the pathogenesis of pituitary tumors, regardless of their hormonal state. This transcription factor is expressed at substantial levels in 96% of tumor cells, on average. Its expression level in pituitary adenomas is significantly higher than in the normal pituitary gland and has no reliable correlation with any other study hormones or Ki-67. In our opinion, NeuroD1’s consistently high expression levels in all pituitary adenoma types make it an attractive potential target for new drugs. If drugs can be designed or screened which reduce NeuroD1 expression to levels near those seen in the normal pituitary gland, such drugs could be vital in the treatment of aggressive neuroendocrine tumors or in the prevention of their reoccurrence.

## SUPPLEMENTARY MATERIALS FIGURES


