# Supplementary material for: Analysis of pituitary adenoma expression patterns suggests a potential role for the NeuroD1 transcription factor in neuroendocrine tumor-targeting therapies
Source: Oncotarget. 2019 Jan 8;10(3):289–312. doi: 10.18632/oncotarget.26513 (PMC6349459; doi:10.18632/oncotarget.26513)
Supplement: Supplementary file 1 [file oncotarget-10-289-s001.pdf]

Analysis of pituitary adenoma expression patterns suggests a potential role for the NeuroD1 transcription factor in neuroendocrine tumor-targeting therapies

SUPPLEMENTARY MATERIALS

Immunohistochemical study

Pituitary gland paraffin sections were deparaffinized with xylene and rehydrated. In order to inactivate endogenous peroxidase, sections were treated with 3% hydrogen peroxide (5 min. at room temperature) and then washed in distilled water. Antigen retrieval was performed with boiling in EDTA (pH 9.0) in a water bath (Thermo Fisher). Then, sections were washed in Tris-Buffer with Tween 20 (2 washes of 5 min. each). Bovine serum incubation was carried out at room temperature, in a container with wet filter paper, for 20 min. Primary antibody incubations were performed at 4°C for 18 hours.

Secondary antibody incubations, with either mouse EnVision™+ Peroxidase System, or rabbit EnVision™+ Peroxidase System (DAKO, Denmark) were performed at 37°C for 30 minutes. Next, sections were washed in Tris-Buffer with Tween 20 (2 washes of 5 min. each). Coloured DAB reaction products were visualized directly by light microscopy. After washing in distilled water, the sections were counterstained with hematoxylin for 2 minutes, dehydrated, and then mounted using a permanent mounting medium (Polystyrol, BioMount, Italy).

For double immunostaining, the deparaffinized and rehydrated pituitary sections were treated with TRIS EDTA (pH 9.0) at 95-98°C in a water bath for 35 minutes and allowed to cool at room temperature for 20 minutes. To reduce non-specific background staining, the tissue specimens were incubated with UltraVBlock for 10 minutes at room temperature. Primary antibody cocktail #1 contained rabbit PRL antibody and mouse NeuroD1 antibody. Primary antibody cocktail #2 contained rabbit GH antibody and mouse NeuroD1 antibody. Each primary cocktail was incubated for 30 minutes at room temperature with MultiVision Polymer Cocktail (Thermo Scientific, UK), which contains anti-rabbit/HRP and anti-mouse/AP. Next, LVBlue and LVRed working solutions were applied, and the sections were incubated for 10 minutes in each reagent. After final washing, the sections were dried and embedded in glycerol. In each cocktail, mouse antibodies were visualized with blue color, and rabbit antibodies visualized with red color.

Confocal laser scanning microscopy

The study was done on deparaffinized and rehydrated sections ranging from 4 to 10 µm in thickness. Heat-induced epitope retrieval was performed with 0.01 M citrate buffer (pH 6.0) under pressure. PBS buffer with Tween 20 was used as a wash buffer. Then, sections were incubated for 30 minutes in blocking solution (PBS containing 2% BSA) at room temperature. After washing, the first primary NeuroD1 antibodies were applied for 1 hour at room temperature. Then, after additional washing, the sections were incubated with an additional primary antibody (GH or prolactin) for 1 hour at room temperature. Alexa Fluor 647®-labeled anti-mouse secondary (Abcam, UK) and Alexa Fluor 488®-labeled anti-rabbit secondary antibodies (Abcam, UK) were used for visualization.

After washing, nuclei in the sections were stained with DAPI (AppliChem). Dako Mounting Medium (DAKO, Denmark) was used for mounting all tissue specimens. As a result, NeuroD1 signal was seen as red fluorescence, and GH or prolactin signals were seen as green fluorescence; contrasted nuclei were seen as blue fluorescence. Preparations were analyzed using the Olympus FV1000D confocal laser scanning microscope. We evaluated the intensity and colocalization of NeuroD1 expression and DAPI fluorescence.

Micrographs of 4 mammosomatotropinomas (5 fields per each sample, at 400x magnification), were analyzed for double staining patterns (GH/NeuroD1 or prolactin/NeuroD1). The co-expression coefficient of hormones and NeuroD1 in the same cells was defined as the ratio of the double-stained cells to the total number of cells, expressed as a percentage. The NeuroD1 expression coefficient was calculated as the ratio of NeuroD1-positive nuclei to DAPI stained nuclei (NeuroD1/DAPI, as a percent). The NeuroD1 expression coefficient and co-expression coefficients (of hormones and NeuroD1) were determined using image analysis software (Image Scope Color M, Russia).

Electron immunocytochemistry

Small (1-2 mm) fragments of tumors were first fixed in PBS-buffered 4% paraformaldehyde solution, containing 0.2% glutaraldehyde, for 1 hr and post-fixed in 1% PBS-buffered OsO_4 solution for 1 hour. Next, specimens were dehydrated, using a series of increasing ethanol concentrations, and embedded in LR White resin (Sigma-Aldrich Inc., St.-Louis, Missouri, USA). The resin was polymerized in tightly-capped gelatin capsules at +52 °C. Ultrathin sectioning (60-80 nm) of embedded specimens was performed using an EM UC7 ultramicrotome (Leica, Germany). Ultrathin sections of

samples were collected on nickel electron microscopy grids. In order to prevent non-specific binding of primary antibodies, the sections on grids were incubated in PBS containing 1% bovine serum albumin (BSA-PBS, Sigma-Aldrich Inc.) at room temperature for 15 min.

In the ultrathin sections, an indirect immunogold labeling procedure was used for the detection of NeuroD1 in mammosomatotropinoma specimens and for the detection of NeuroD1 and GH in somatotropinoma specimens. Mouse monoclonal anti-NeuroD1 antibody (clone ab60704, Abcam, United Kingdom), diluted 1:400, was used for NeuroD1 detection. Rabbit polyclonal anti-GH antibody (BioGenex, USA), diluted 1:100, was used for GH detection. As secondary antibodies, we used goat-anti mouse antibody conjugated to 10nm colloidal gold (Sigma-Aldrich Inc., St.-Louis, Missouri, USA) and goat-anti rabbit antibody conjugated to 5nm colloidal gold (Sigma-Aldrich Inc., St.-Louis, Missouri, USA), both diluted 1:100.

Sections on grids were incubated in phosphate buffer containing primary antibodies for 1 hour, and subsequently washed in PBS buffer containing 0.05% TWEEN 20. Next, grids were incubated with secondary antibodies for 1 hour and washed, in the same manner, with PBST. Finally, sections on grids were contrasted in aqueous solutions of uranyl acetate, followed by lead citrate. Electron-microscopic examination was carried out using a JEM 1011 transmission electronic microscope (JEOL, Tokyo, Japan) equipped with a high-resolution digital camera (Morada, Olympus, Japan).

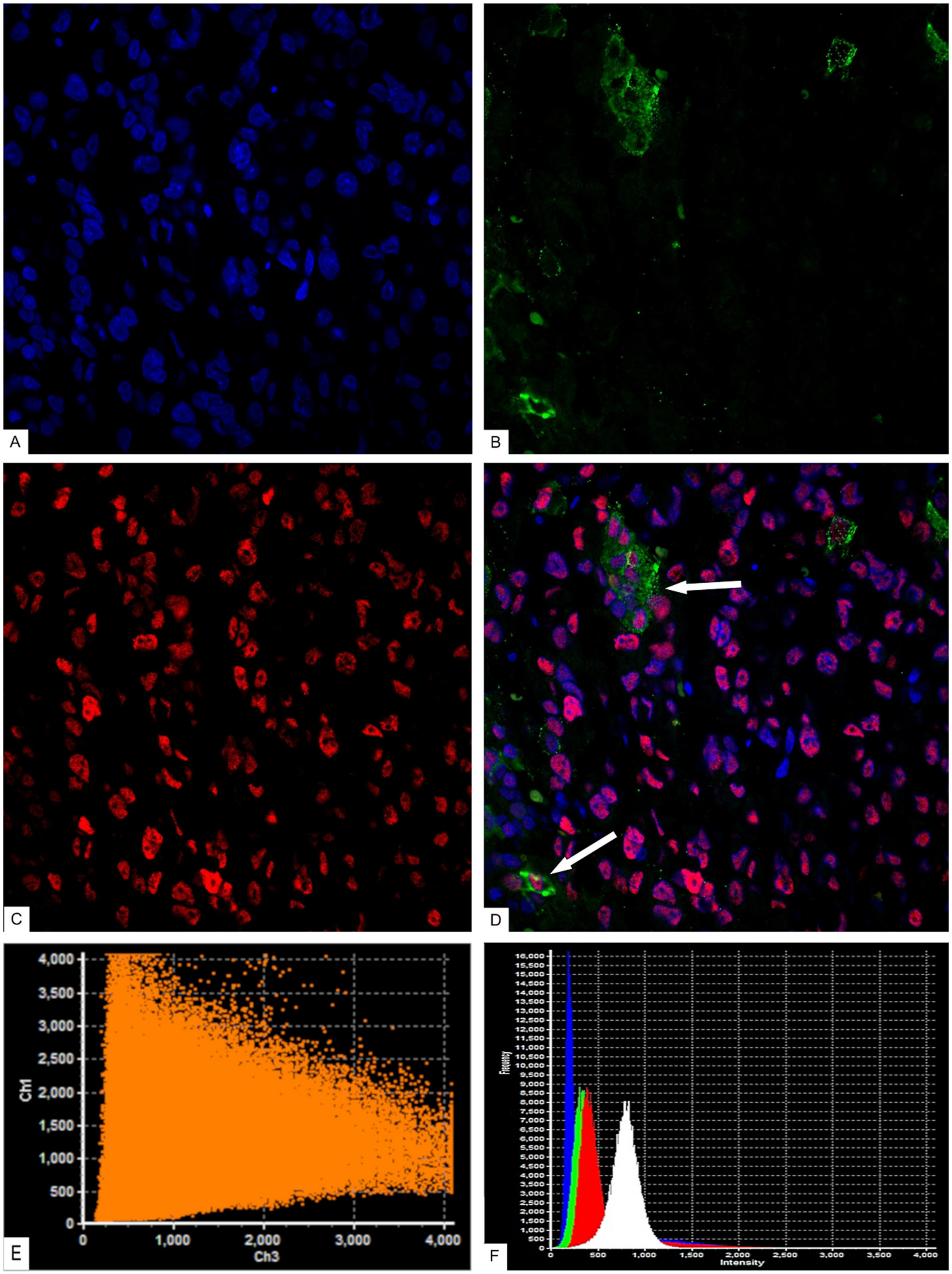

Supplementary Figure 1: Confocal laser scanning microscopy, mammosomatotropinoma №2. **(A)**: blue fluorescence of cell nuclei (DAPI); **(B)**: green fluorescence of Prolactin; **(C)**: red fluorescence of NeuroD1; **(D)**: overlay image **(A, B, C)**. NeuroD1(pink fluorescence)/Prolactin (green fluorescence) same cell co-expression is seen in 16% of the cells (indicated by arrows); × 600; **(E)**: scatterplot of blue (DAPI, Ch1) and red (Neuro D1, Ch 3) pixel intensities of tumor cell nuclei; **(F)**: intensity histogram of red (Neuro D1), green (Prolactin), and blue (DAPI) fluorescence. White channel: light microscopy; *(Continued)*

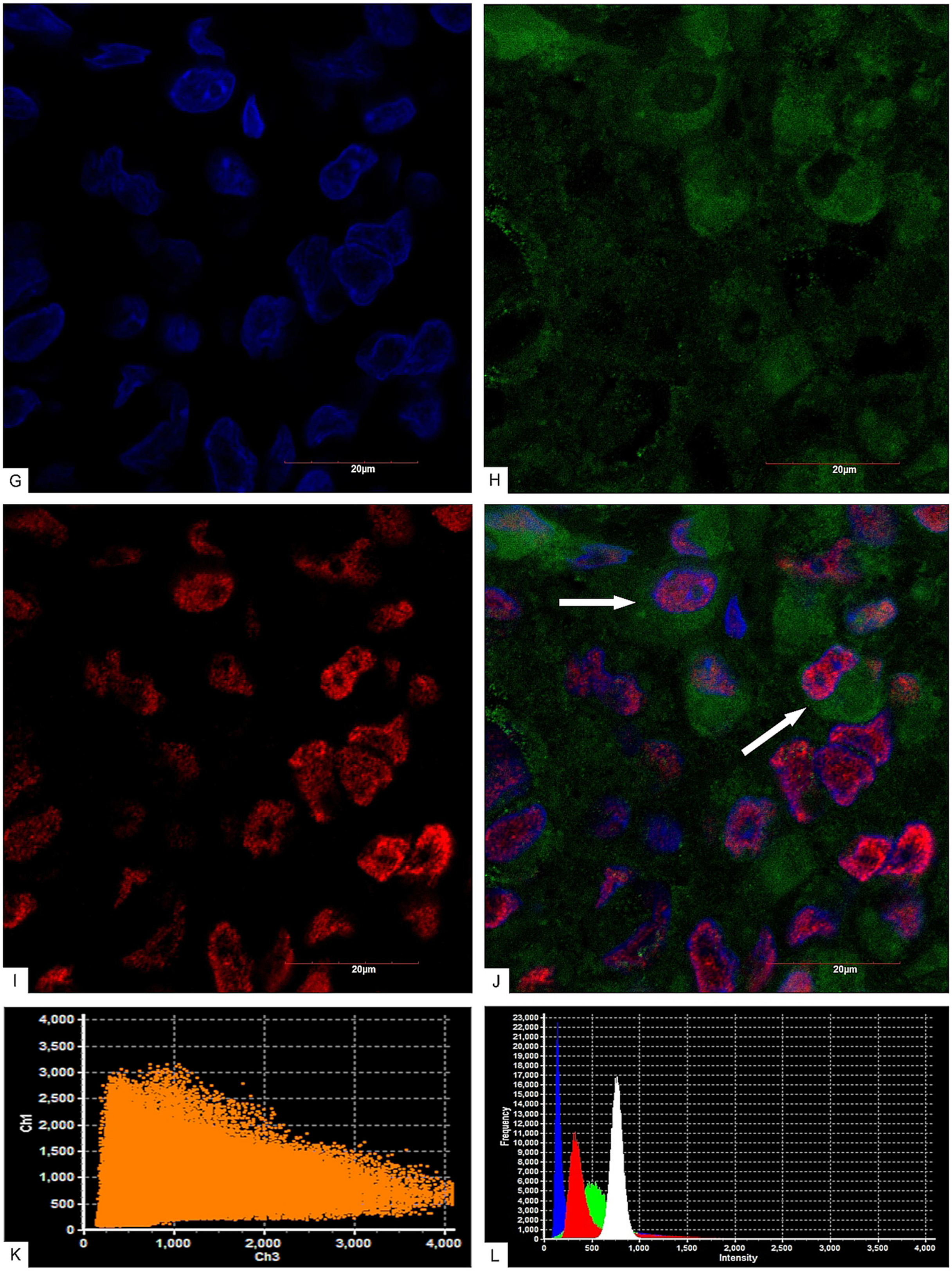

Supplementary Figure 1 ***(Continued)*****: Confocal laser scanning microscopy, mammosomatotropinoma №2. (G)**: blue fluorescence of cell nuclei (DAPI); **(H)**: green fluorescence of Growth hormone; **(I)**: red fluorescence of NeuroD1; **(J)**: overlay image **(G, H, I)**. NeuroD1(pink fluorescence)/Growth hormone (green fluorescence) same cell co-expression is seen in 86% of the cells (indicated by arrows); × 1800; **(K)**: scatterplot of blue (DAPI, Ch1) and red (Neuro D1, Ch 3) pixel intensities of tumor cell nuclei; **(L)**: intensity histogram of red (Neuro D1), green (Growth hormone), and blue (DAPI) fluorescence. White channel: light microscopy.

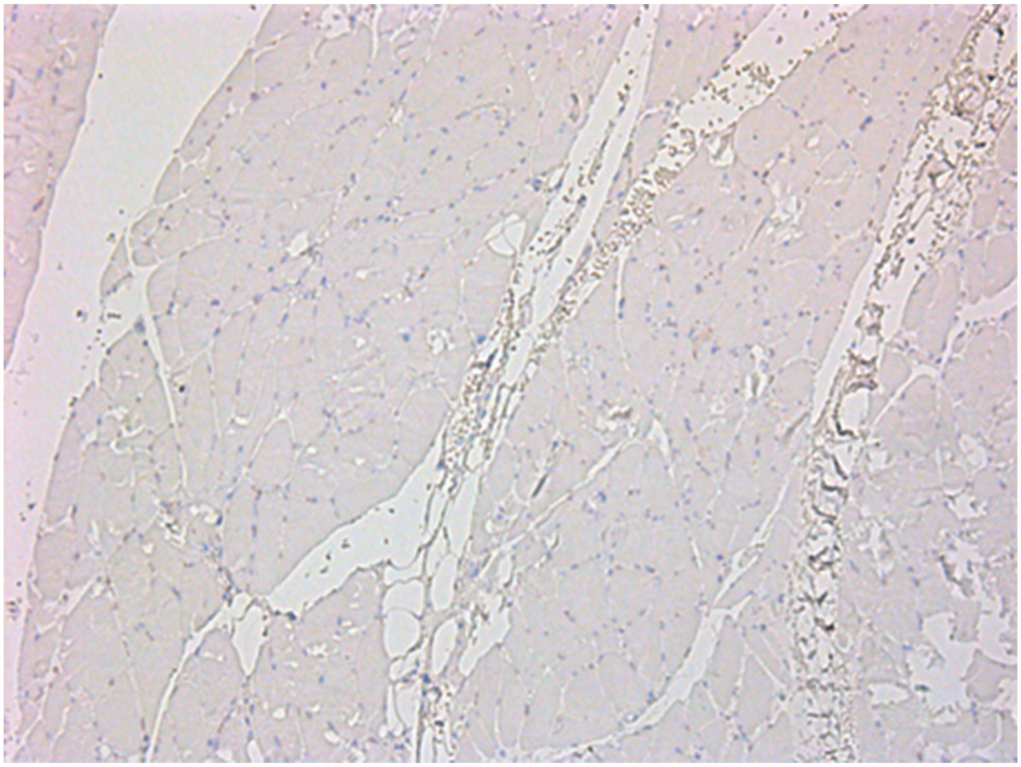

Supplementary Figure 2: Skeletal muscle section stained with primary antibodies to Neuro D1, as negative control, x100.
